# Supplementary material for: De novo histidine biosynthesis protects Mycobacterium tuberculosis from host IFN-γ mediated histidine starvation
Source: Commun Biol. 2021 Mar 25;4:410. doi: 10.1038/s42003-021-01926-4 (PMC7994828; doi:10.1038/s42003-021-01926-4)
Supplement: Supplementary file 2 — Supplementary Information [file 42003_2021_1926_MOESM2_ESM.pdf]

SUPPLEMENTARY INFORMATION

De novo histidine biosynthesis protects *Mycobacterium tuberculosis* from host IFN-γ mediated histidine starvation

Abhisek Dwivedy, Anam Ashraf, Bhavya Jha, Deepak Kumar, Nisheeth Agarwal and Bichitra K. Biswal*

*Corresponding author

This file includes:

Supplementary Figures

Figure 1- The Δ*hisD* fails to grow within infected cell lines and show minimal infiltration into the spleen of infected B6 mice.

Figure 2- Purification and antibody generation data for *Mtb* Histidine biosynthesis enzymes.

Figure 3- The dynamics and inhibition of histidine catabolism enzymes in in vivo and ex vivo *Mtb* infection scenario.

Figure 4- The quality control, alignment and feature assessment of RNASeq readcounts.

Figure 5- Pathways populated with the differentially expressed genes in the KEGG database.

Figure 6- Pathways populated with the differentially expressed genes in the Reactome database.

Figure 7- GO terms related to Immune functions populated with the differentially expressed genes in ClueGO.

Figure 8- An interaction network depicting the genes populating the GO terms for Immune functions.

Figure 9- The IFN-γ signalling pathway from the Reactome database.

Figure 10- The IFN-γ signalling pathway from the WikiPathways.

Figure 11- The IL-6 signalling pathway from the Reactome database.

Figure 12- The possible transcription factors of HAL and HDC and their expression in *Mtb* infected B6 mice lungs.

Figure 13- A cartoon representation of the hypothesized IFN-γ pathway regulating the expression of HAL and HDC.

Figure 14- Activation of infected macrophages with CD8 or naïve CD4 T cells fail to restrict the growth of *ΔhisD*.

Figure 15- Specificity of IFN-γ in bacillary clearance of Δ*hisD*.

Figure 16- The internal Pathophysiology of *Mtb* infected IFN-$\gamma^{-/-}$ mice.

Figure 17- Change is expression of HAL and HDC following T cell activation and IFN-γ supplementation.

Figure 18- The dynamics of free histidine in *Mtb* infected wild type B6 and B6 IFN-$\gamma^{-/-}$ mice.

Figure 19- Reproducibility, linearity and quality control analysis of SRM method development.

Figure 20- The dynamics of histidine metabolites in *Mtb* infected wild type B6 mice.

Figure 21- Linearity and quality control analysis of SRM method development for intracellular free tryptophan quantitation in mice lung lysates.

Figure 22- Full Western blots for images presented in Figures 2A and B.

Figure 23- Full Western blots for images presented in Figures 3B and 5C.

Figure 24- Full Western blots for images presented in Supplementary Figures 1B, 2D and 17.

Supplementary Tables

Table 1- Data Quality Summary for RNA Seq

Table 2- Data QC Statistics for RNA Seq

Table 3- Read Alignment Summary for RNA Seq

Table 4- Feature Assignment Summary for ReadCounts

Table 5- List of Genes used to generate the interaction network in figure 3A

Table 7- Reagent/Resource/Tools

Table 7- Softwares and Parameters for RNA Seq data processing and analysis

SUPPLEMENTARY FIGURES

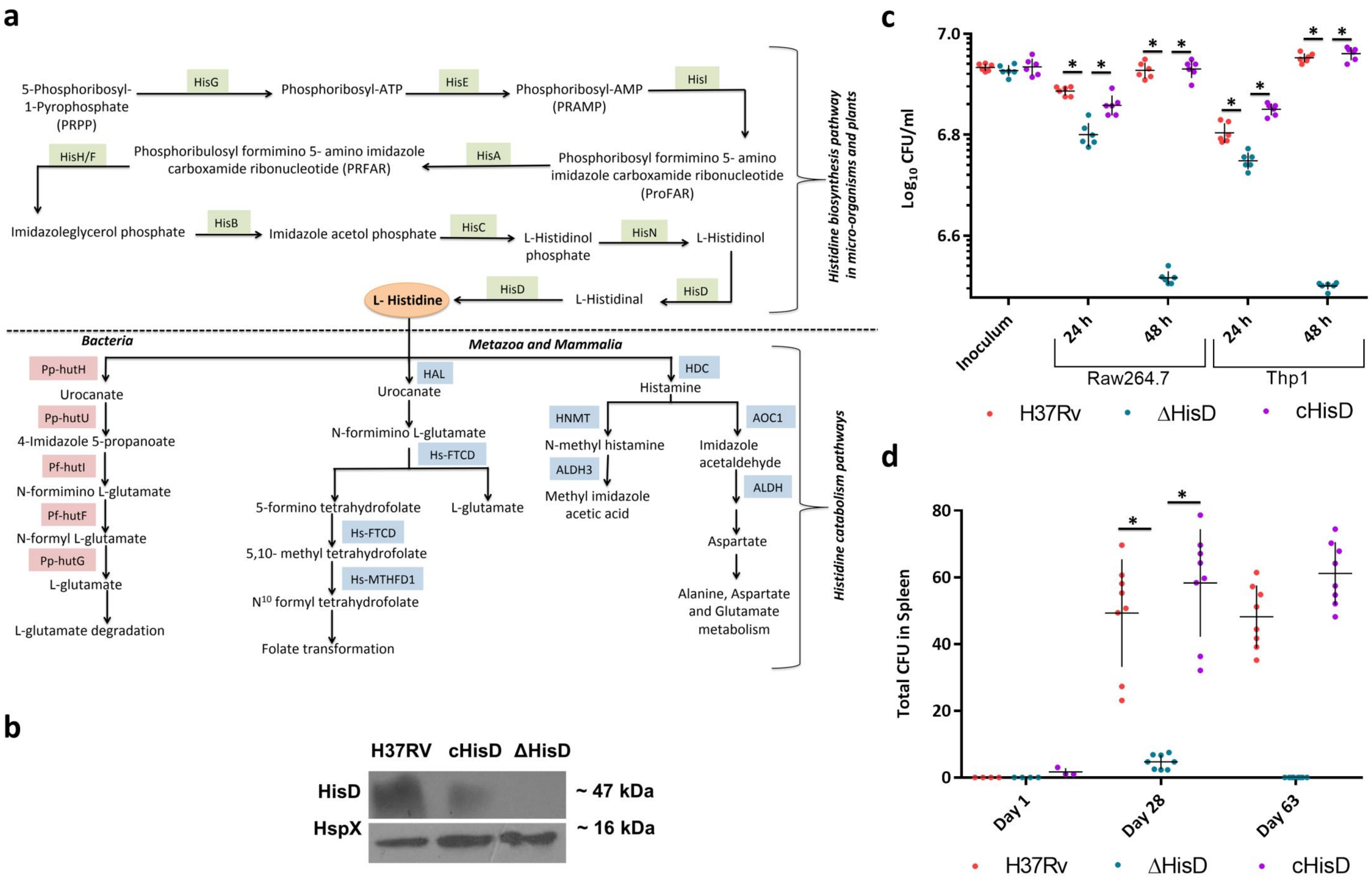

Supplementary Figure 1: The Δ*hisD* fails to grow within infected cell lines and show minimal infiltration into the spleen of infected B6 mice. **(a)** The biosynthesis and catabolism of L-Histidine. **(b)** A comparison of HisD expressions in H37Rv, Δ*hisD* and c*hisD* demonstrating the expression of HISD protein in c*hisD* as compared to Δ*hisD*. (The immunoblots are representative of 3 samples each). **(c)** A decline in cfu of Δ*hisD* (5 folds) over a course of 48 h post infection in cells lines (Raw 264.7 & Thp1); completely rescued in c*hisD* hint at the indispensability of HISD in ex vivo infection (n=6 independently cultured cell samples; mean & SEM; *P-value<0.05). **(d)** Minimal infiltration of bacilli into spleens of the B6 mice infected by H37Rv, Δ*hisD* and c*hisD* was observed over a period of 9 weeks. H37Rv and c*hisD*, however, showed significantly higher cfu counts as compared to Δ*hisD* (n=8 individual mice per time point; mean and SEM; *P-value<0.05).

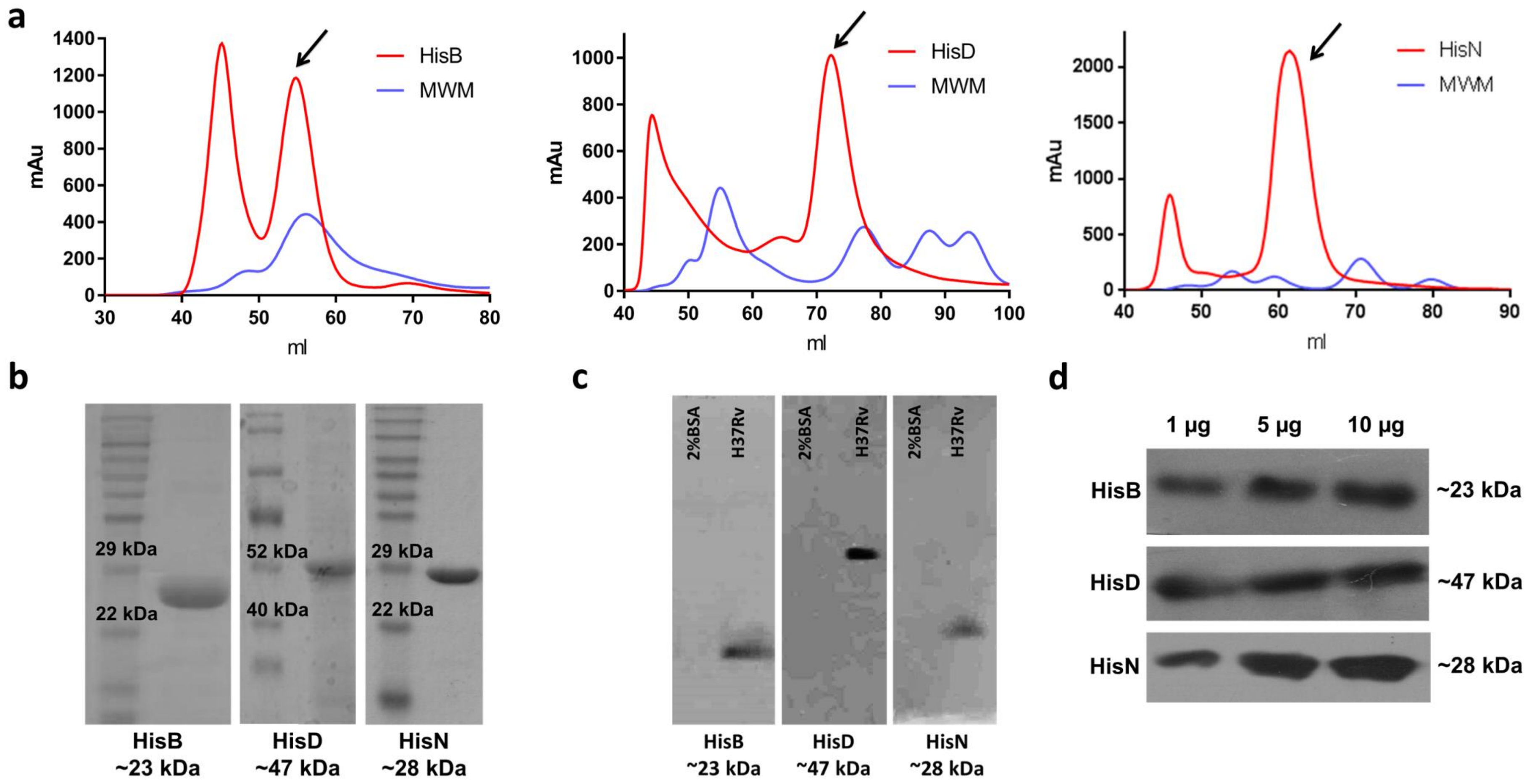

Supplementary Figure 2: Purification and antibody generation data for *Mtb* Histidine biosynthesis enzymes. (a) Size exclusion chromatogram profiles for HisB (left panel), HisD (center panel) and HisN (right panel), shown in red curves with respective protein peaks highlighted with arrows. The blue curves denote molecular weight markers. Markers for HisB and HisDa re of sizes 669 kDa, 440 kDa, 158 kDa, 75 kDa and 44 kDa respectively from left to right. Markers for HisN are of sizes 75 kDa, 44 kDa, 25.6 kDa and 13.7 kDa respectively from left to right. HisD and HisN exist as dimers in solutions while HisB exists as a 24-mer complex. **(b)** Peaks highlighted in Supplementary Figure 3 as observed on SDS PAGE

showing single bands depicting high degree of purity and homogeneity. **(c)** Immunoblots showing specificity of anti-sera containing polyclonal antibodies against respective proteins against whole cell lysate of H37Rv. 2% BSA used as a negative control. (The immunoblots are representative of 3 samples each). **(d)** Immunoblots showing sensitivity of anti-sera containing polyclonal antibodies against respective purified proteins. (The immunoblots are representative of 3 samples each).

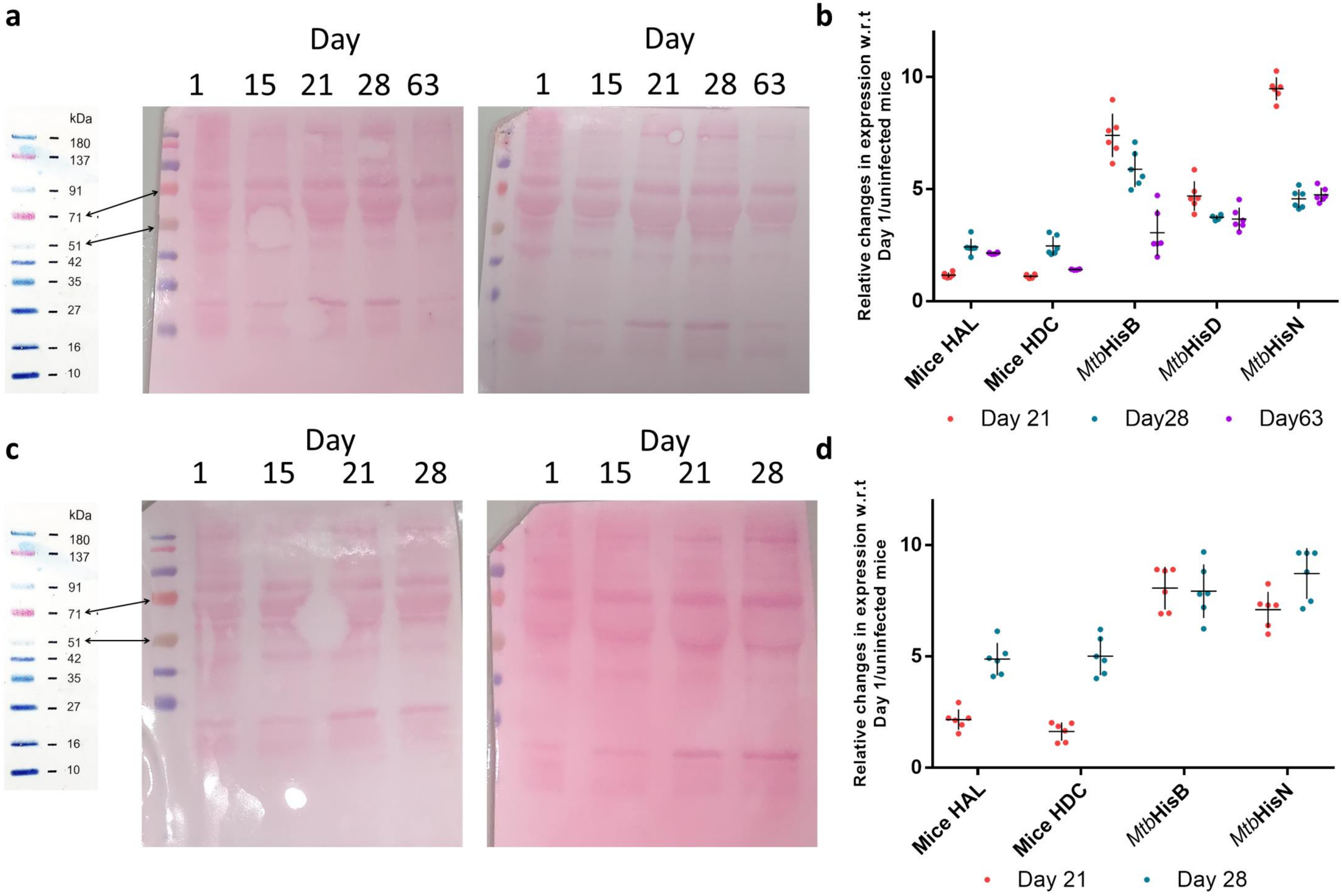

a
Day
1 15 21 28 63
Day
1 15 21 28 63
kDa
180
137
91
71
51
42
35
27
16
10
b
Relative changes in expression w.r.t Day 1/uninfected mice
0
5
10
Mice HAL
Mice HDC
MtbHisB
MtbHisD
MtbHisN
Day 21
Day28
Day63
c
Day
1 15 21 28
Day
1 15 21 28
kDa
180
137
91
71
51
42
35
27
16
10
d
Relative changes in expression w.r.t Day 1/uninfected mice
0
5
10
Mice HAL
Mice HDC
MtbHisB
MtbHisN
Day 21
Day 28

Supplementary Figure 3: The dynamics and inhibition of histidine catabolism enzymes in in vivo and ex vivo Mtb infection scenario. (a) Representative Ponceau stained immunoblots of H37Rv infected B6 lungs (day 1, 15, 21, 28 and 63). **(b)** Changes in expression levels (ELISA) of host histidine sequestering enzymes (HAL & HDC) and Mtb histidine biosynthesis enzymes (HISB, HISD & HISN) on day 21, day 28 and day 63 post infection with H37Rv as compared to day 1 (n=6 samples obtained from individual mice per time point; mean & SEM). **(c)** Representative Ponceau stained immunoblots of ΔhisD infected B6 lungs (day 1, 15, 21 and 28). **(d)** Changes in expression levels (ELISA) of host histidine sequestering enzymes (HAL & HDC) and Mtb histidine biosynthesis enzymes (HISB, HISD & HISN) on day 21 and day 28 post infection with ΔhisD as compared to day 1 (n=6 samples obtained from individual mice per time point; mean & SEM).

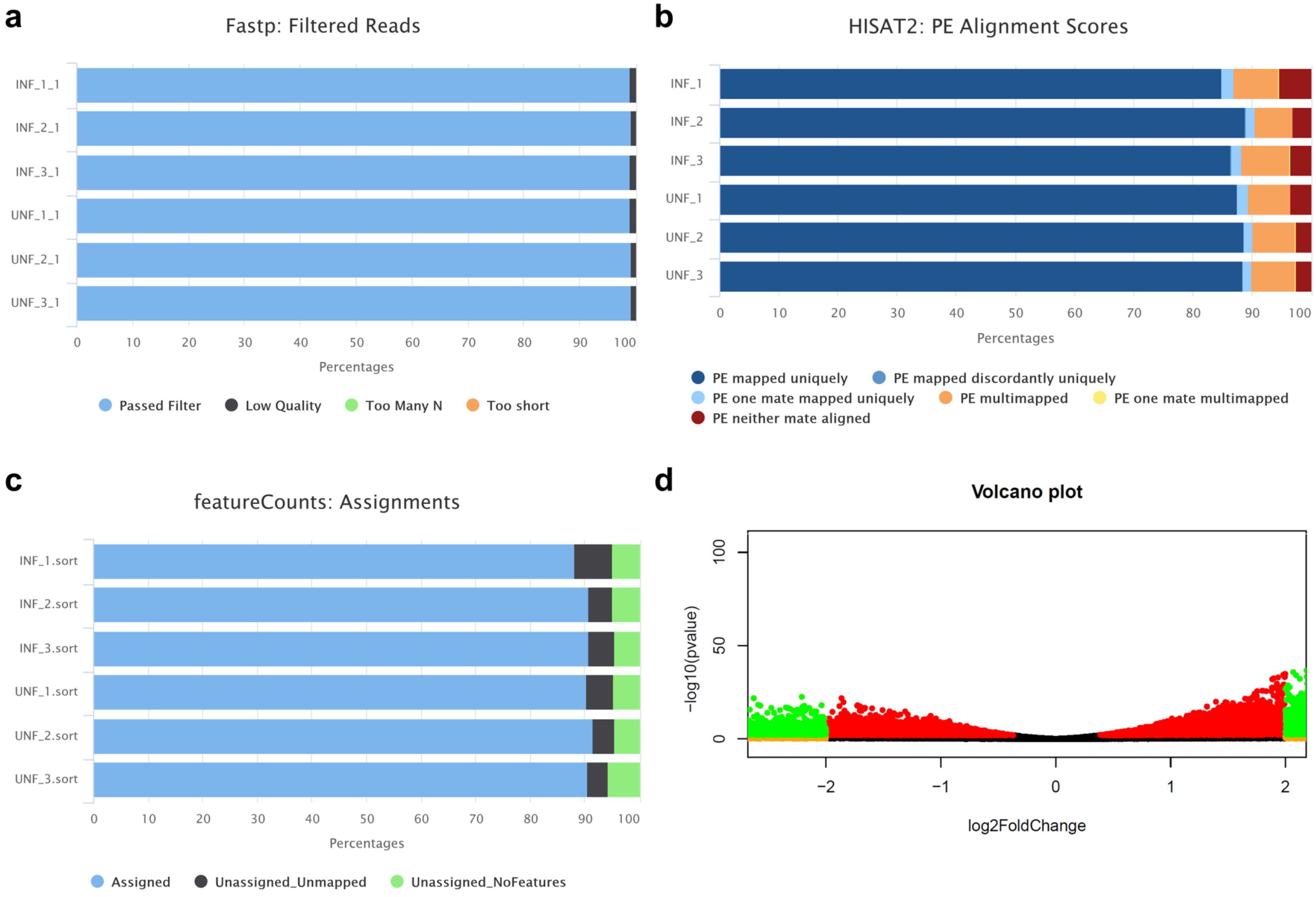
a
Fastp: Filtered Reads
INF_1_1
INF_2_1
INF_3_1
UNF_1_1
UNF_2_1
UNF_3_1
0 10 20 30 40 50 60 70 80 90 100
Percentages
Passed Filter
Low Quality
Too Many N
Too short
b
HISAT2: PE Alignment Scores
INF_1
INF_2
INF_3
UNF_1
UNF_2
UNF_3
0 10 20 30 40 50 60 70 80 90 100
Percentages
PE mapped uniquely
PE mapped discordantly uniquely
PE one mate mapped uniquely
PE multimapped
PE one mate multimapped
PE neither mate aligned
c
featureCounts: Assignments
INF_1.sort
INF_2.sort
INF_3.sort
UNF_1.sort
UNF_2.sort
UNF_3.sort
0 10 20 30 40 50 60 70 80 90 100
Percentages
Assigned
Unassigned_Unmapped
Unassigned_NoFeatures
d
Volcano plot
−log10(pvalue)
0
50
100
−2 −1 0 1 2
log2FoldChange

Supplementary Figure 4: The quality control, alignment and feature assessment of RNASeq readcounts. (a) Quality control analyses of the filtered reads >95% high quality reads for all samples. **(b)** Approximately 90% of the read counts from all samples mapped uniquely with the reference genome of C57BL6. **(c)** Over 85% of the read counts from all samples show perfectly assigned features from the reference genome following alignment. **(d)** Distribution of the differentially expressed genes. (UNF denotes Uninfected; INF denotes H37Rv infected)

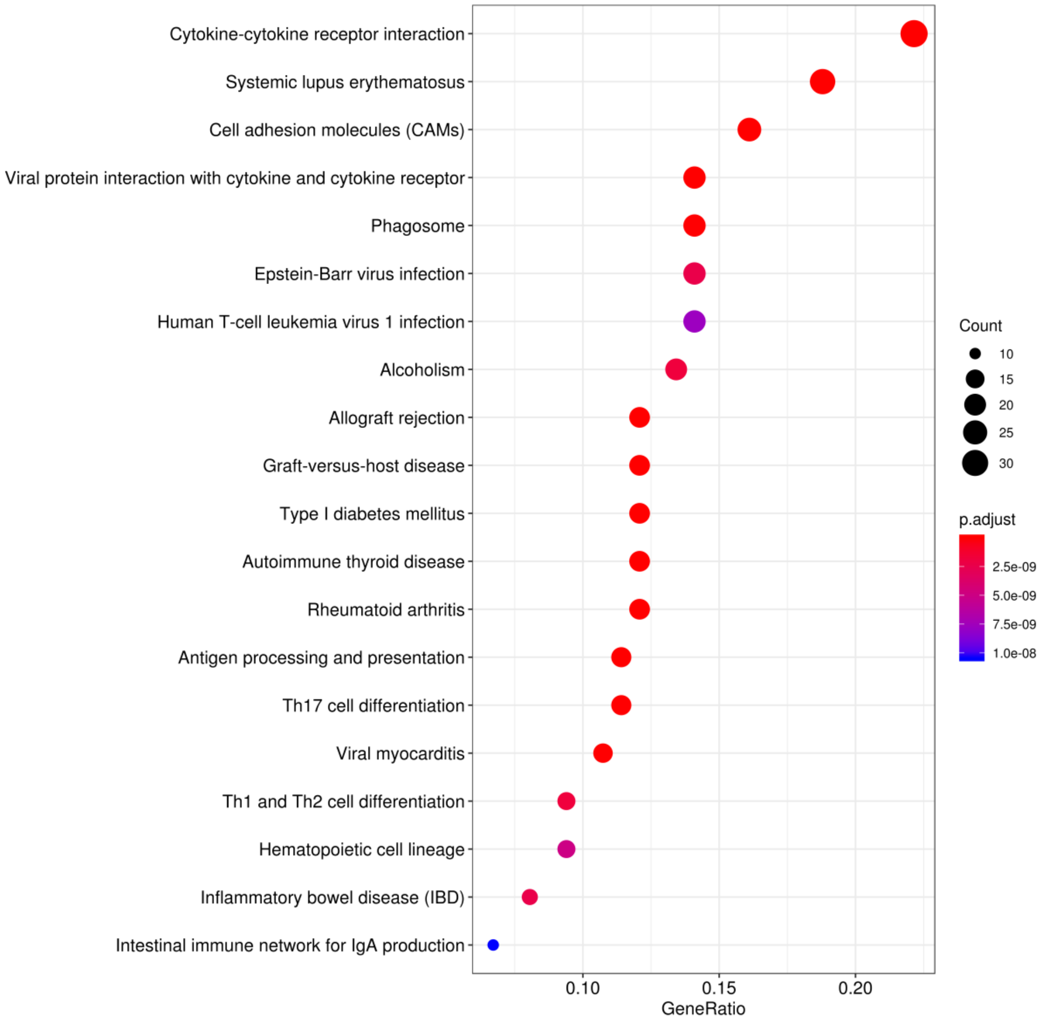

Supplementary Figure 5: Pathways populated with the differentially expressed genes in the KEGG database. A significant proportion of the pathways populated belong to the host immune system.

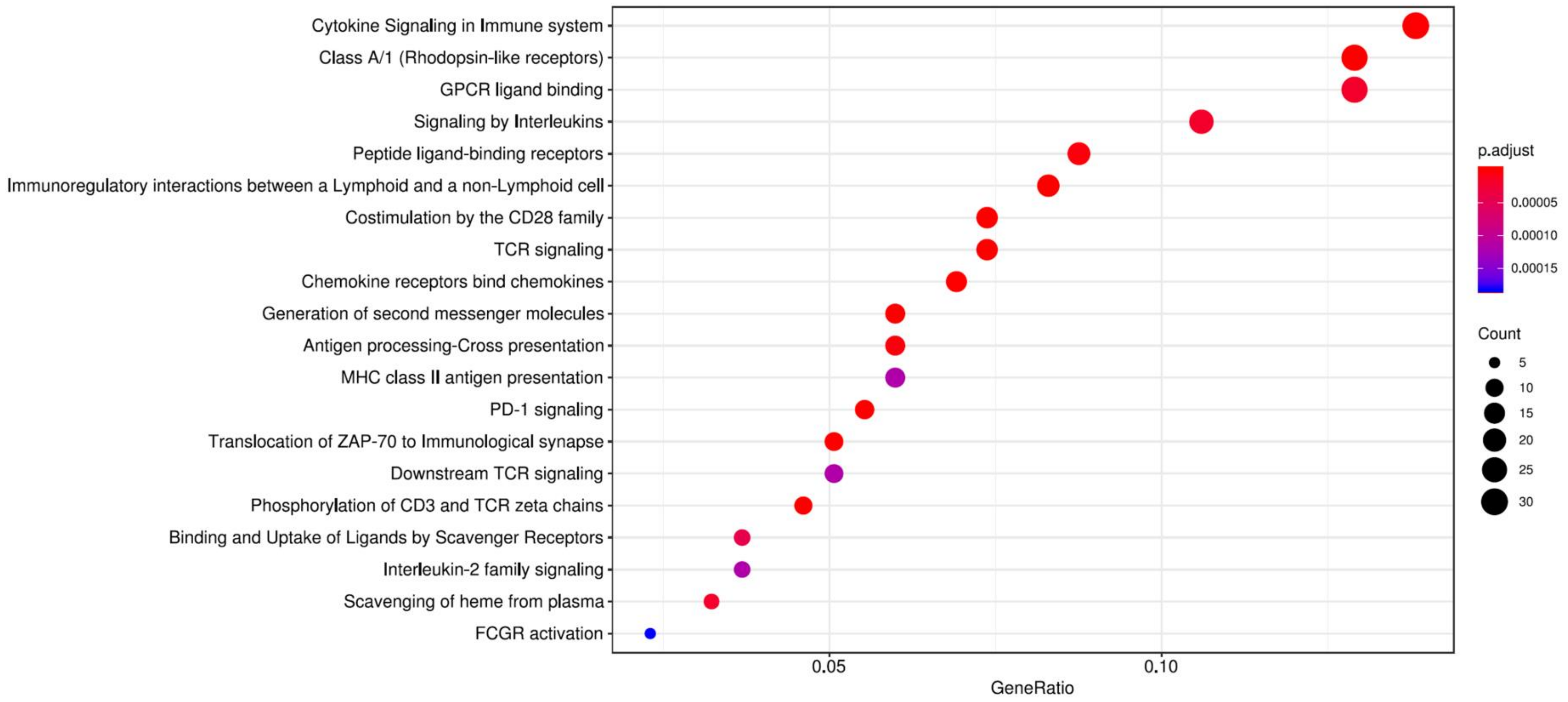

Supplementary Figure 6: Pathways populated with the differentially expressed genes in the Reactome database. Pathways related to cytokine signalling, interleukin signalling and TCR signalling in particular are significantly populated.

a

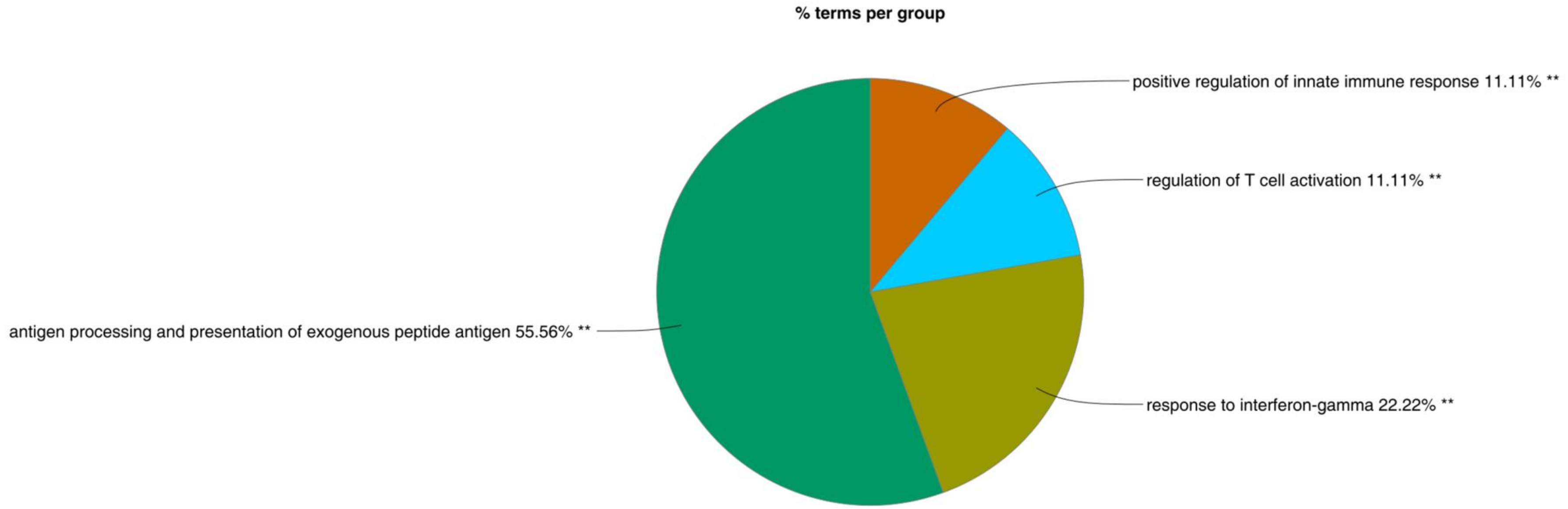

b

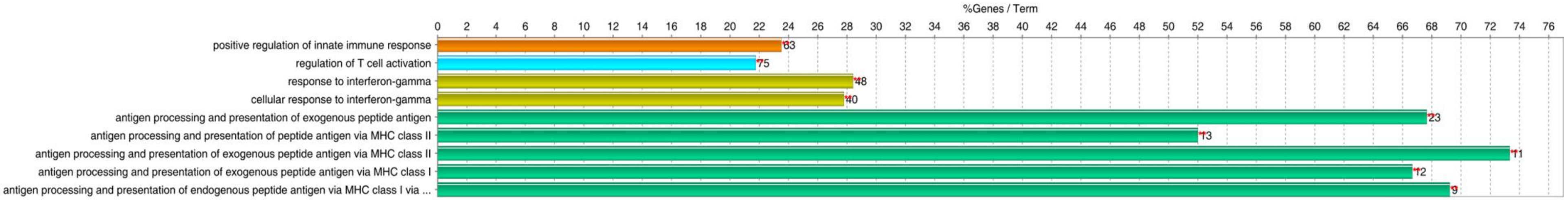

Supplementary Figure 7: GO terms related to Immune functions populated with the differentially expressed genes in ClueGO. (a) Pie chart and **(b)** bar plots. A significant percentage of these genes populate GO terms involved in cellular and other responses to IFN-γ signalling.

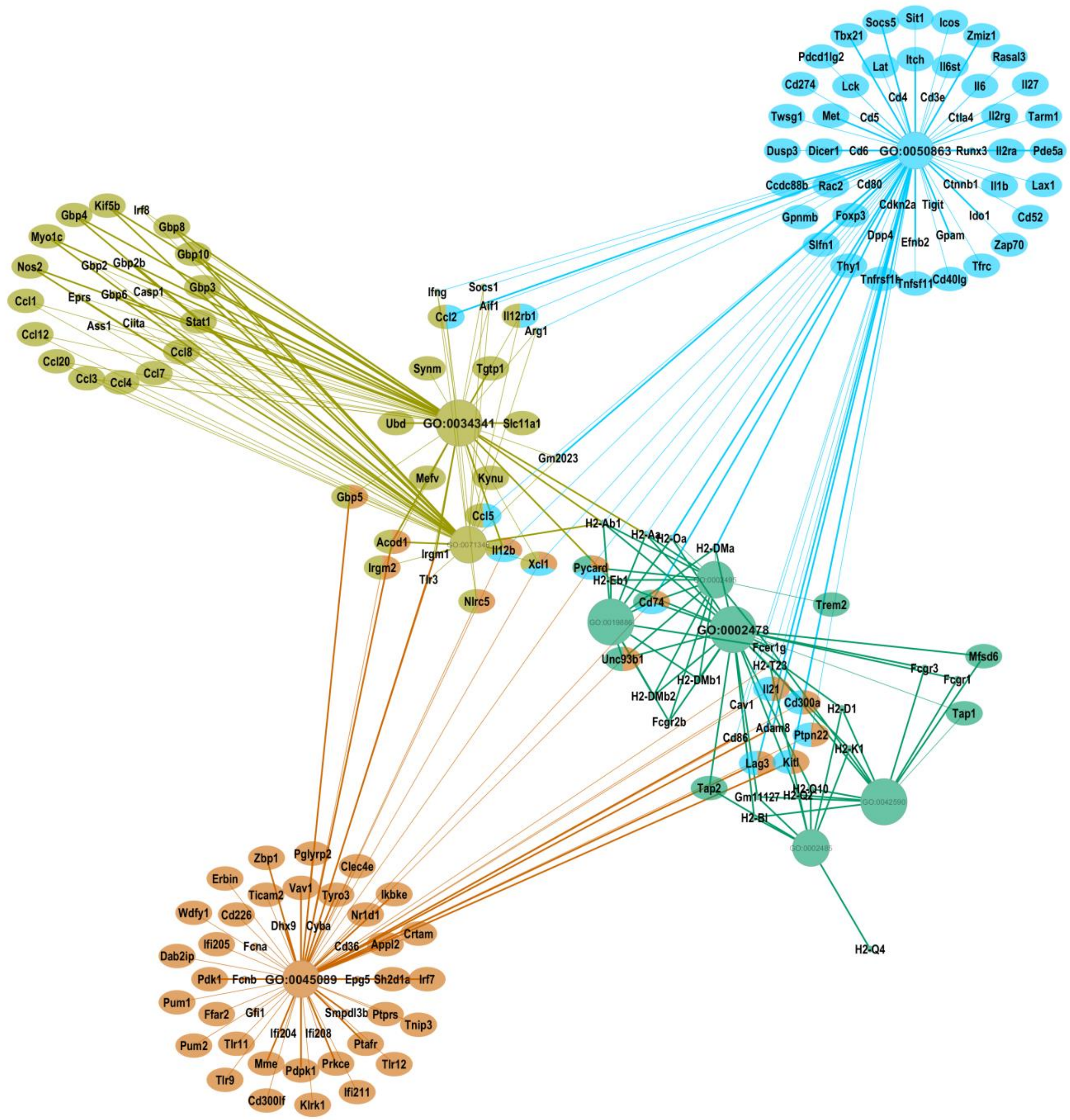

Supplementary Figure 8: An interaction network depicting the genes populating the GO terms for Immune functions. GO:0045089 (positive regulation of innate immune response), GO:0050863 (regulation of T cell activation), GO:0034341 (response to interferon-gamma) and GO:0002478(antigen processing and presentation of exogenous peptide antigen.

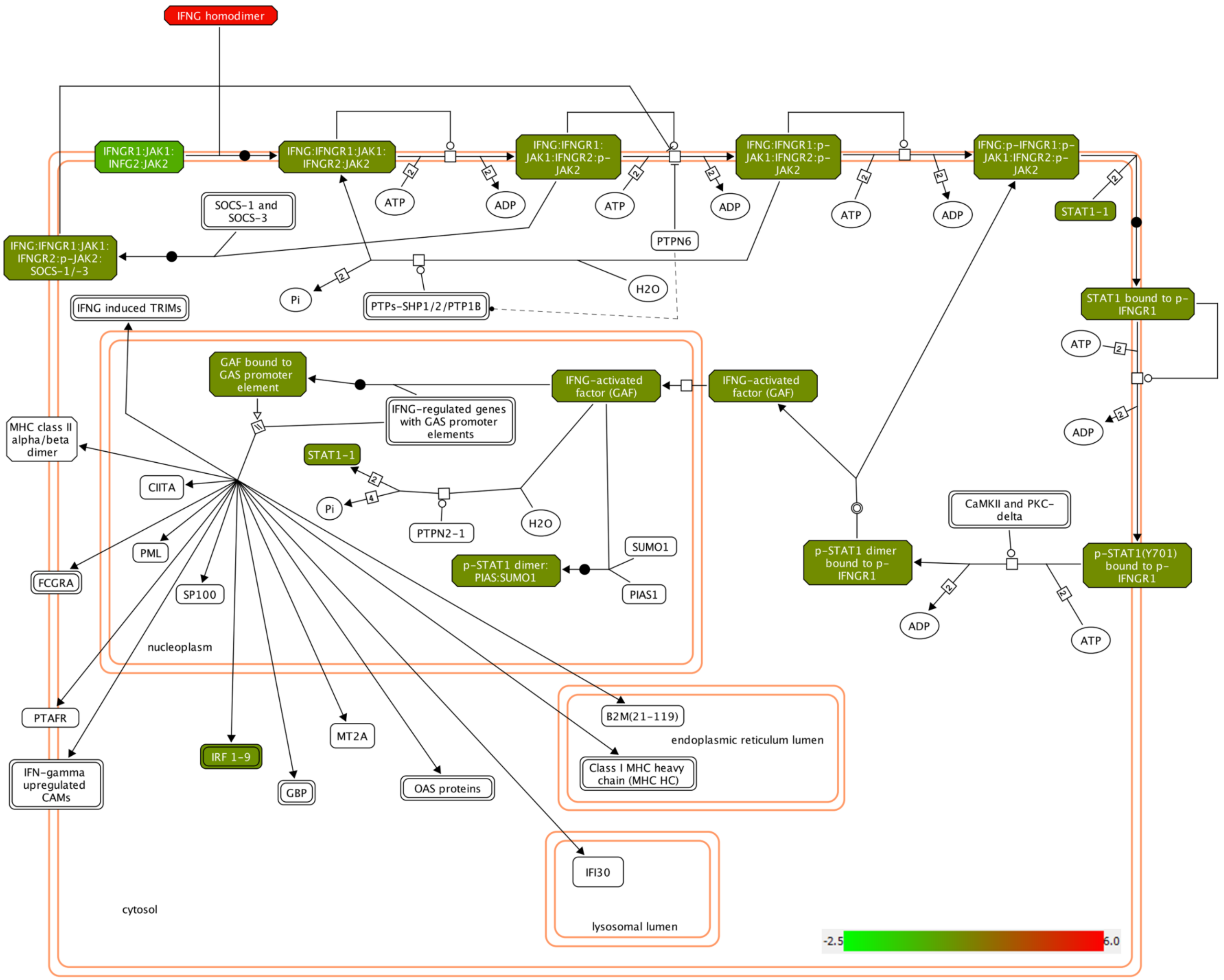

Supplementary Figure 9: The IFN-γ signalling pathway from the Reactome database. Individual genes and/or clusters are overlaid with their respective fold change values from the differentially expression (Fold changes represented in colour variations).

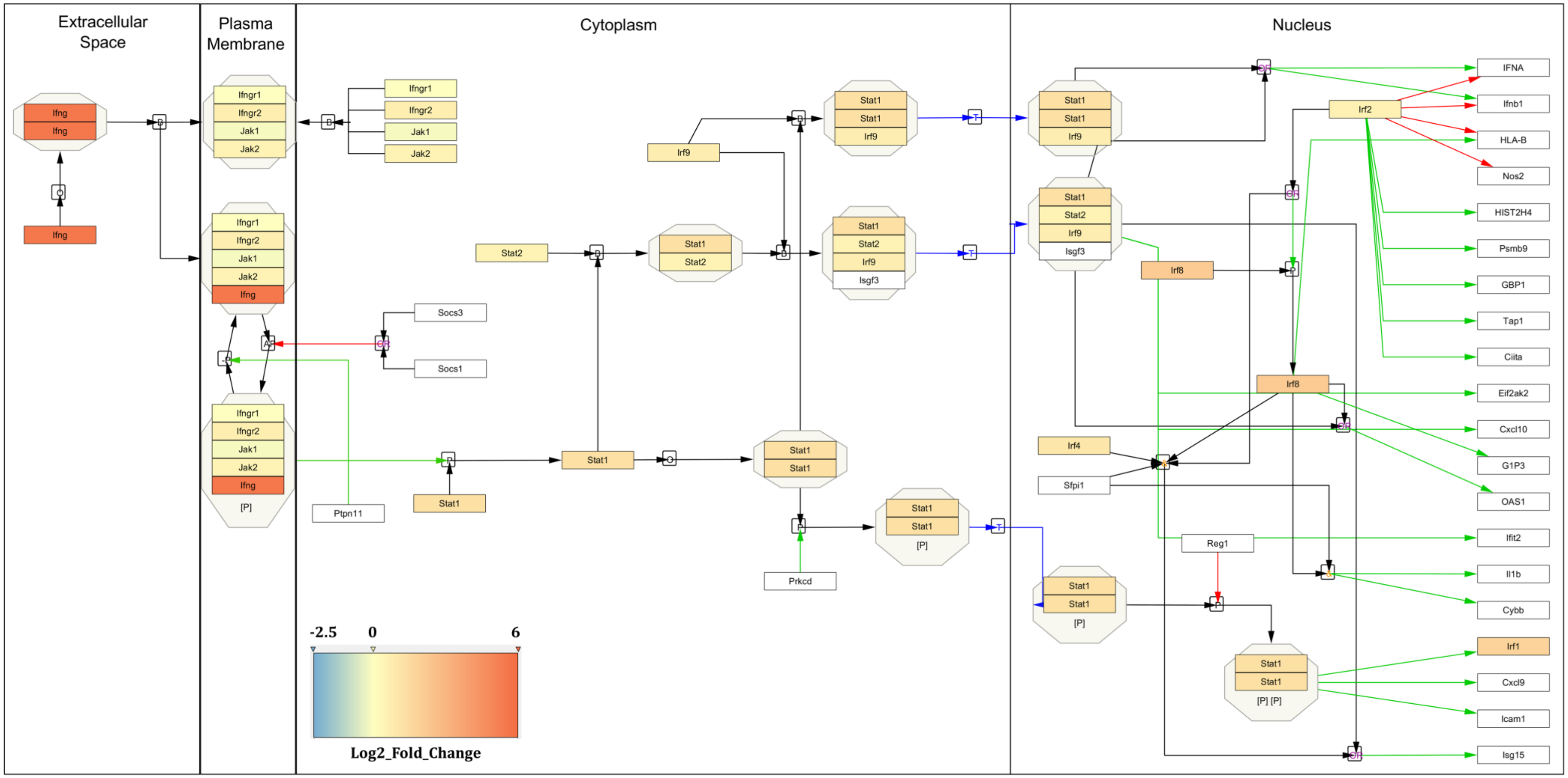

Supplementary Figure 10: The IFN-γ signalling pathway from the WikiPathways. Individual genes are overlaid with their respective fold change values from the differentially expression (Fold changes represented in colour variations).

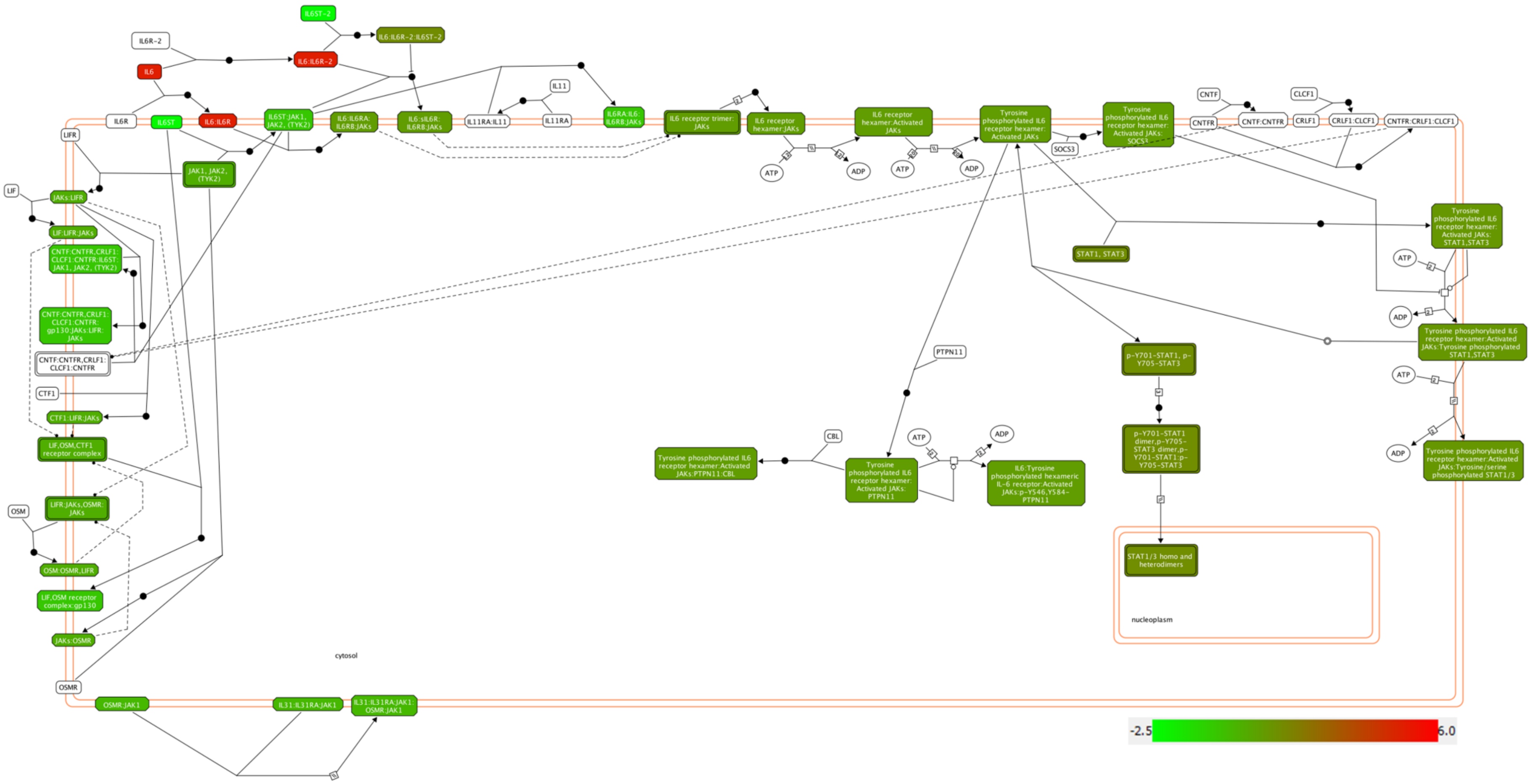

Supplementary Figure 11: The IL-6 signalling pathway from the Reactome database. Individual genes and/or clusters are overlaid with their respective fold change values from the differentially expression (Fold changes represented in colour variations).

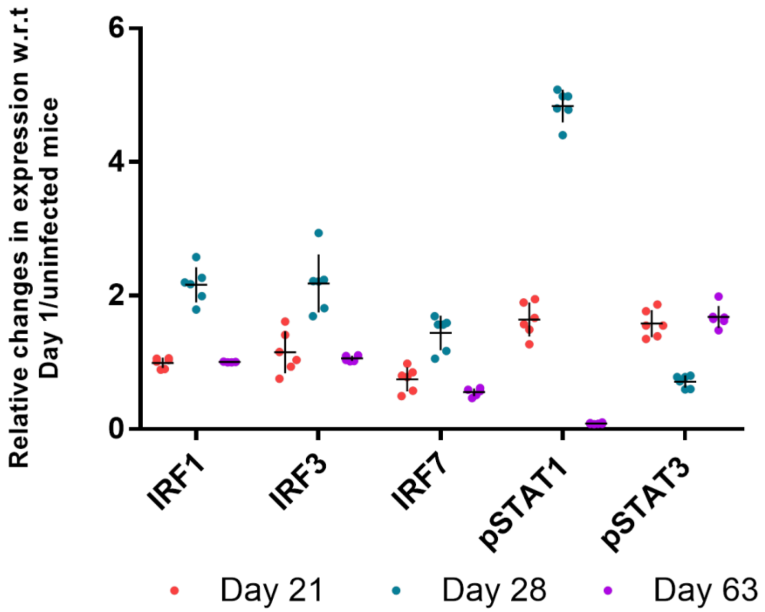

Supplementary Figure 12: The possible transcription factors of HAL and HDC and their expression in *Mtb* infected B6 mice lungs. Changes in expression levels (ELISA) of select key mediators from the IFN-γ downstream signalling involved in upregulation of HAL and HDC on day 21, day 28 and day 63 post infection as compared to day 1 (n= 6 samples obtained from individual mice per time point; mean & SEM).

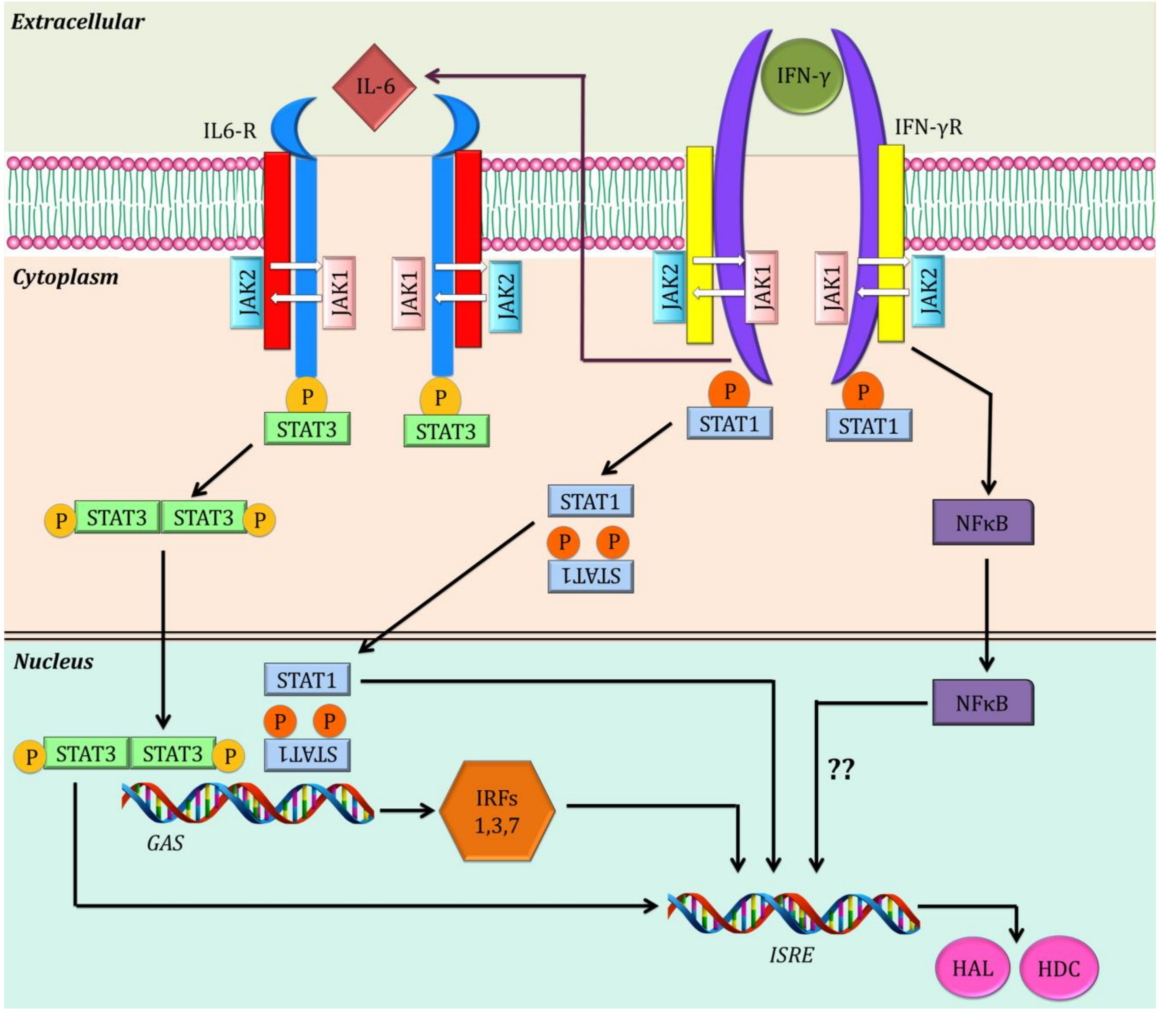

Supplementary Figure 13: A cartoon representation of the hypothesized IFN-γ pathway regulating the expression of HAL and HDC. The signalling follows either through the direct (JAK-STAT1) and/or the indirect (IL-6 mediated JAK-STAT3) downstream signals. The mode of regulation by NFκB remains unclear.

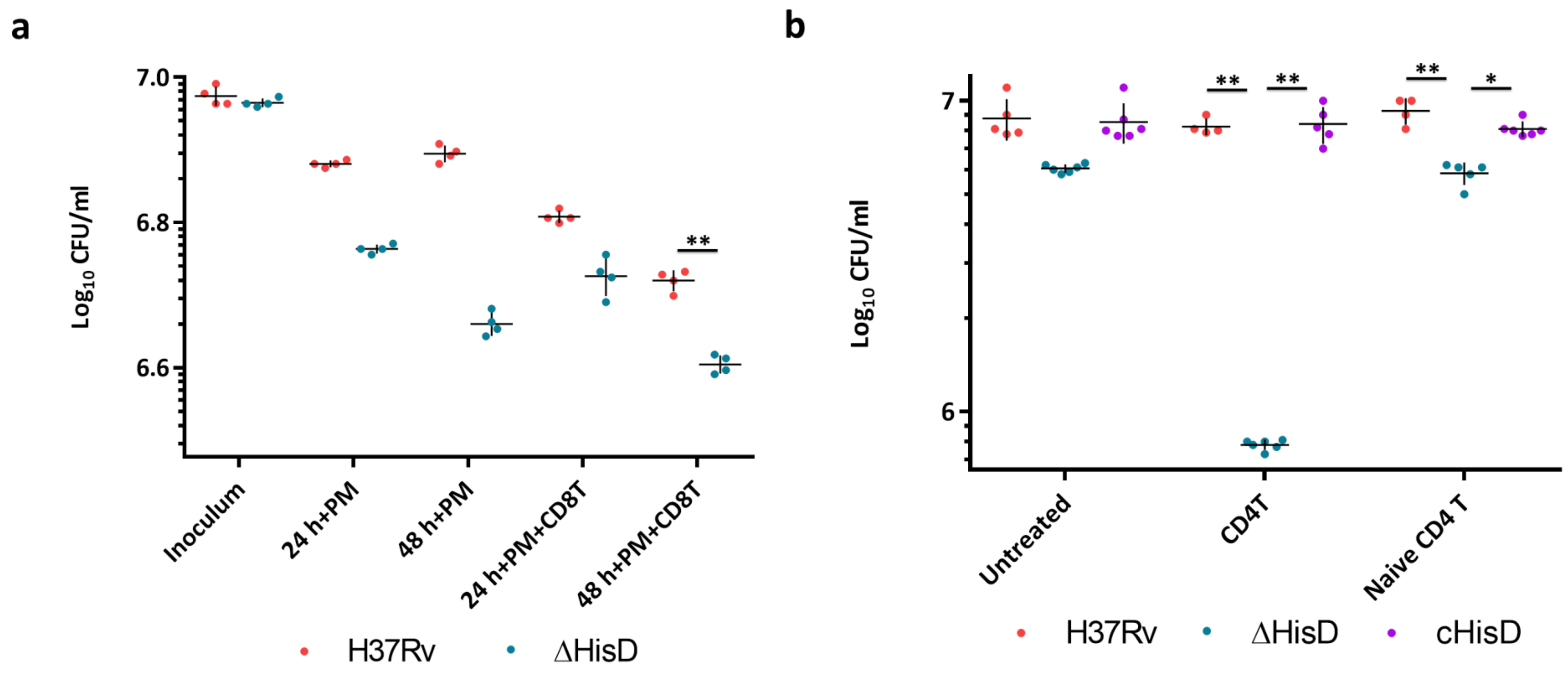

Supplementary Figure 14: Activation of infected macrophages with CD8 or naïve CD4 T cells fail to restrict the growth of Δ*hisD*. (a) Cfu counts for Δ*hisD* show no significant reductions, 48 h posts infection as compared to H37Rv following CD8 T cell activation (n=4 independently acquired and cultured primary cells; mean and SEM; **P-value<0.005). **(b)** Activation of infected primary macrophages with naïve CD4 T cells fail to decrease the Δ*hisD* cfu significantly 48 h post infection as observed in cases of primed CD4 T cells mediated activation (See Fig. 4D). (n=6 independently acquired and cultured primary cells; mean and SEM; **P-value<0.005).

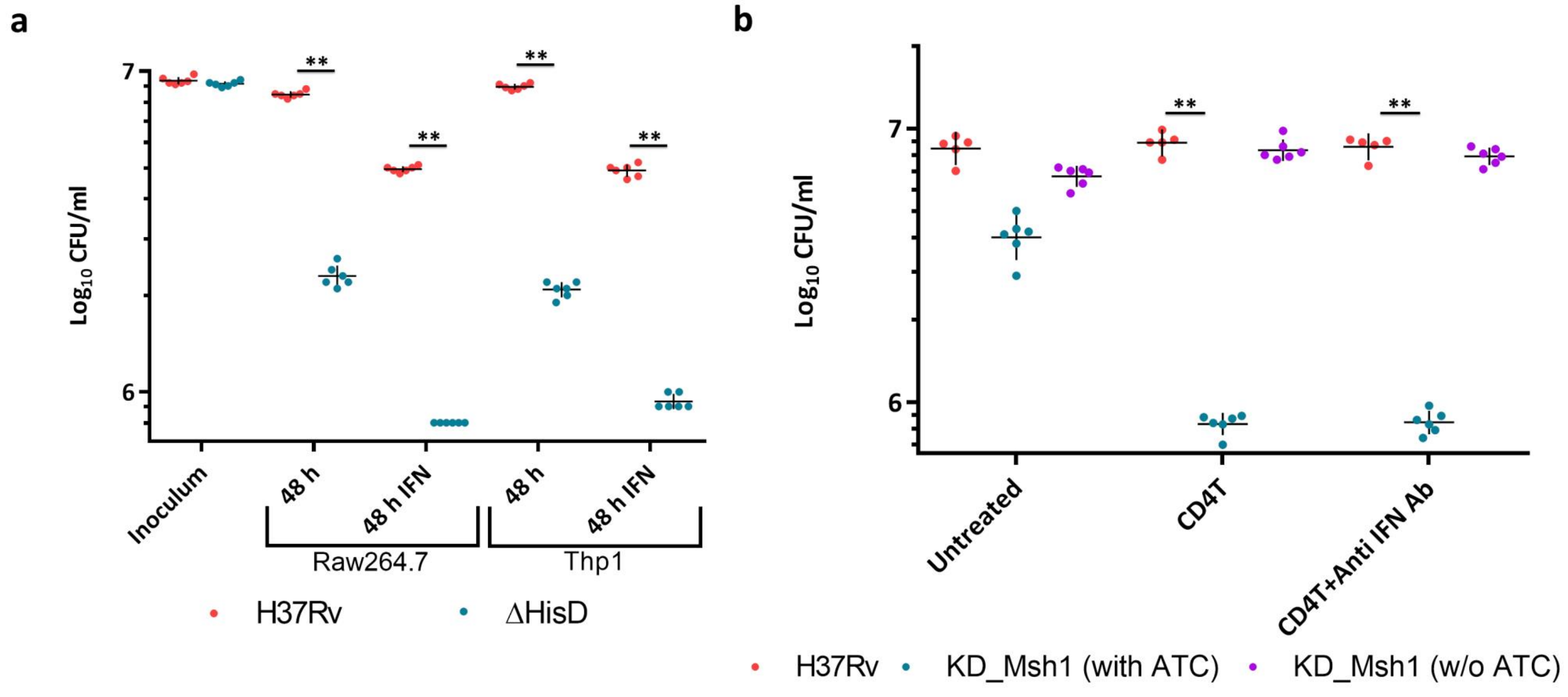

Supplementary Figure 15: Specificity of IFN-γ in bacillary clearance of Δ*hisD*. (a) External supplementation of IFN-γ post infection onto infected cell lines leads to decrease in the cfu counts of Δ*hisD*. (n=6 independently cultured cell samples; mean and SEM; **P-value<0.005). **(b)** An Msh1 knockdown strain of H37Rv shows attenuated growth when infected in macrophages when co-cultured with CD4 T cells. The growth profile remains unchanged despite treatment with antibodies against IFN-γ. (n=6 independently acquired and cultured primary cells; mean and SEM; **P-value<0.005)

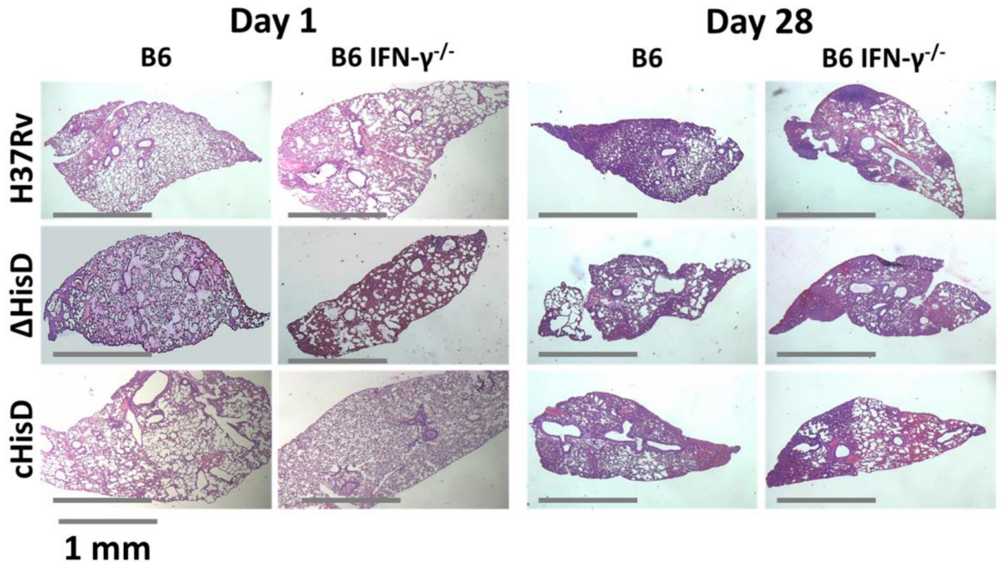

Supplementary Figure 16: The internal Pathophysiology of *Mtb* infected IFN-γ$^{-/-}$ mice. The internal pathology of the lungs suggests higher degree of damage in H37Rv and c*hisD* infected B6 mice on day 28; a comparable extent of internal lung lesions and reduced air spaces was observed in B6 IFN-γ$^{-/-}$ mice infected with H37Rv, Δ*hisD* and c*hisD* on day 28 (Pictures are representative of 6 individual mice lungs samples).

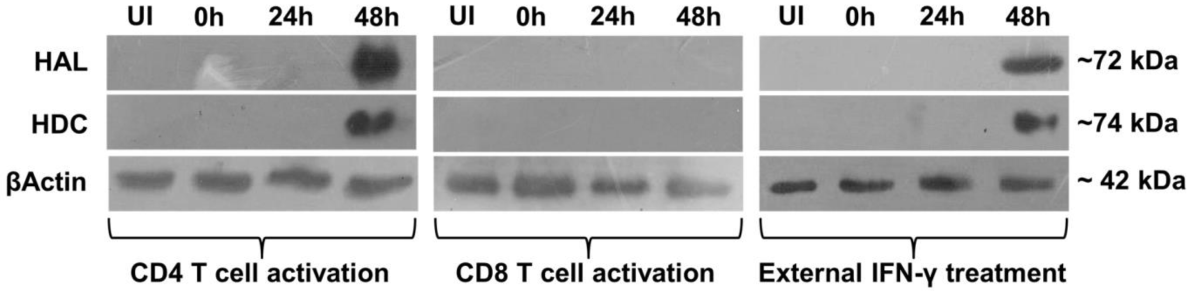

Supplementary Figure 17: Change is expression of HAL and HDC following T cell activation and IFN-γ supplementation. Immunoblots depicting an up regulation of HAL and HDC 48 h post infection in infected primary murine macrophages, following activation specifically with CD4 T cells and external IFN- γ, but not with CD8 T cells. (UI- uninfected; Immunoblots are representative of 6 samples acquired from independent experimental setups for each time point).

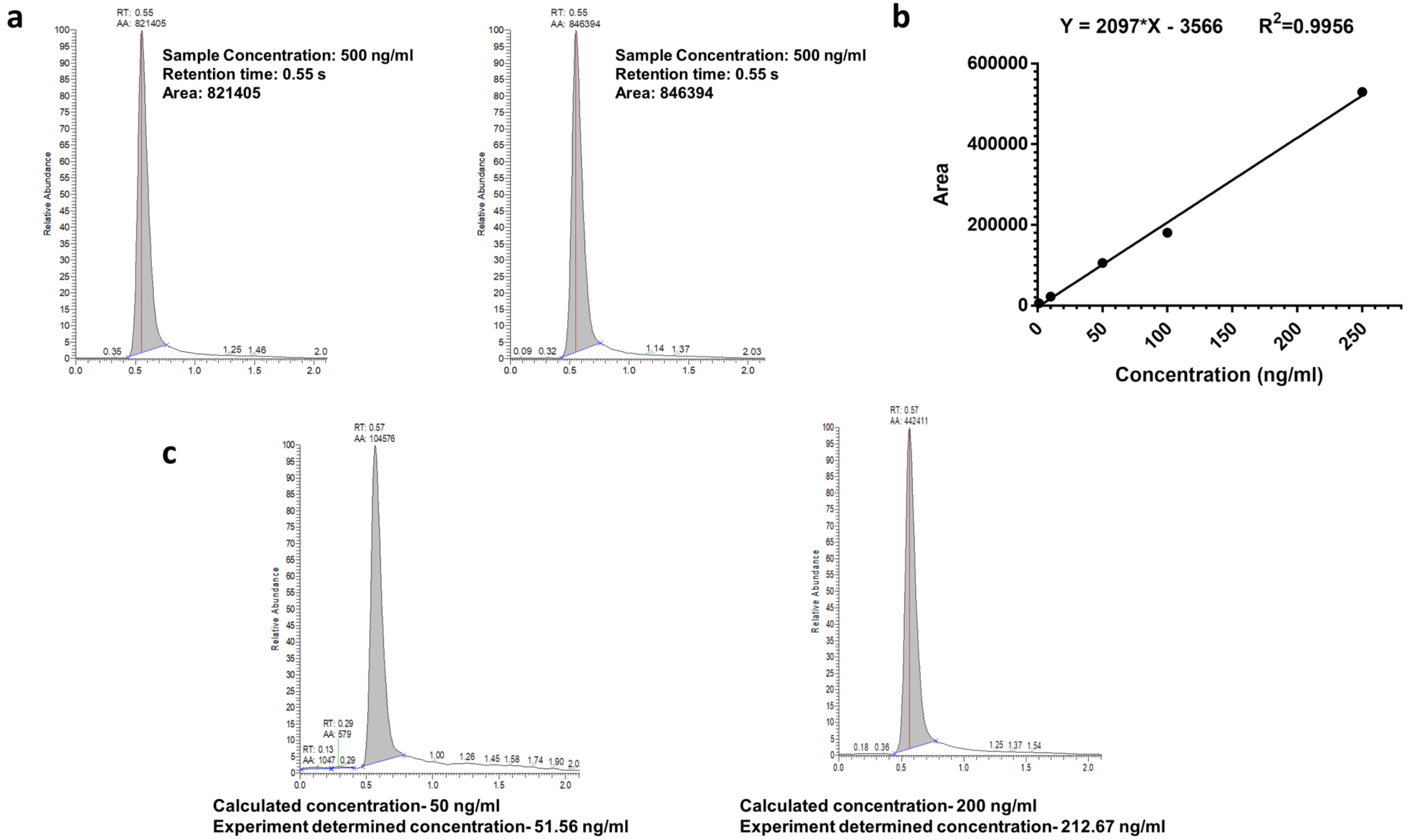

Supplementary Figure 18: Reproducibility, linearity and quality control analysis of SRM method development for intracellular free histidine quantitation in mice lung lysates. (a) Reproducibility test revealing the similarity in retention time and area corresponding to the histidine peak in two independent infusions for a concentration of 500 ng (x-axis represents time). **(b)** Linearity curve generated for pure histidine, concentrations

ranging from 1 to 250 ng ml^{-1}. **(c)** Quality control test for two different concentrations of pure histidine and their respective quantitation from the linearity plot suggest a high degree of confidence between the calculated and experimentally determined values of concentrations (x-axis represents time).

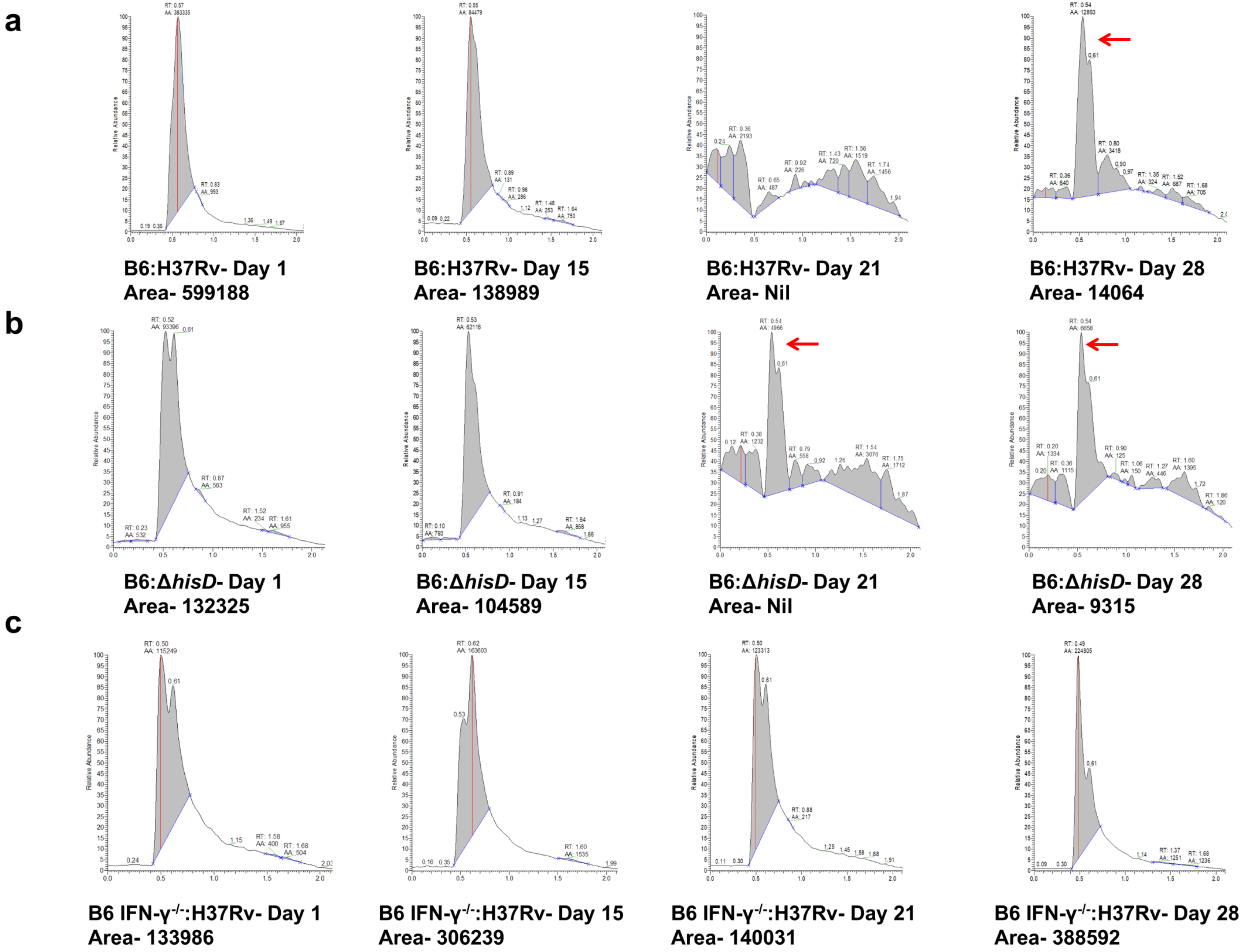
a
B6:H37Rv- Day 1
Area- 599188
B6:H37Rv- Day 15
Area- 138989
B6:H37Rv- Day 21
Area- Nil
B6:H37Rv- Day 28
Area- 14064
b
B6:ΔhisD- Day 1
Area- 132325
B6:ΔhisD- Day 15
Area- 104589
B6:ΔhisD- Day 21
Area- Nil
B6:ΔhisD- Day 28
Area- 9315
c
B6 IFN-γ-/-:H37Rv- Day 1
Area- 133986
B6 IFN-γ-/-:H37Rv- Day 15
Area- 306239
B6 IFN-γ-/-:H37Rv- Day 21
Area- 140031
B6 IFN-γ-/-:H37Rv- Day 28
Area- 388592
Relative Abundance

Supplementary Figure 19: The dynamics of intracellular free histidine in *Mtb* infected B6 wild type and IFN-$\gamma^{-/-}$ mice. (a) LC-MS peaks for free histidine in B6 mice lungs infected with H37Rv on day 1, day 15 and day 28. No histidine was detected on day 21 (Graphs are representative of 6 samples for each time point). **(b)** LC-MS peaks for free histidine in B6 mice lungs infected with Δ*hisD* on day 1, day 15 and day 28. No histidine was detected on day 21 (Graphs are representative of 6 samples for each time point). **(c)** LC-MS peaks for free histidine in B6 IFN-$\gamma^{-/-}$ mice lungs infected with H37Rv on day 1, day 15, day 21 and day 28 (Graphs are representative of 6 samples for each time point) (All x-axes represent time).

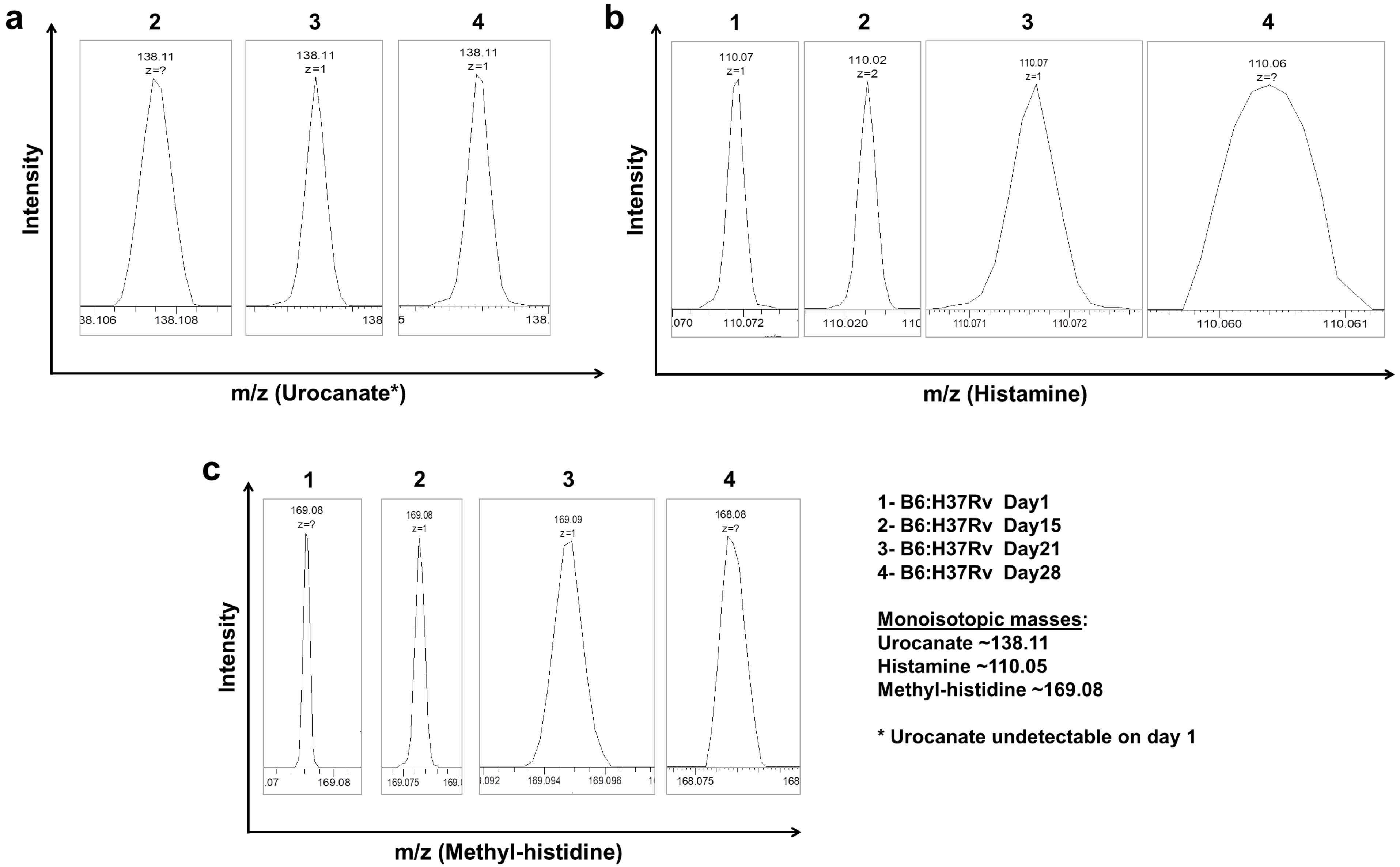

Supplementary Figure 20: The dynamics of histidine metabolites in *Mtb* infected wild type B6 mice. (a) Comparative analysis of urocanate intensities for histidine normalized samples at 3 different time points of infection. Of note, no urocanate was detected on day 1 (Graphs are representative of 6 samples for each time point). **(b)** Comparative analysis of histamine intensities for histidine normalized samples at 4 different time

points of infection (Graphs are representative of 6 samples for each time point). **(c)** Comparative analysis of Methyl-histidine intensities for histidine normalized samples at 4 different time points of infection (Graphs are representative of 6 samples for each time point).

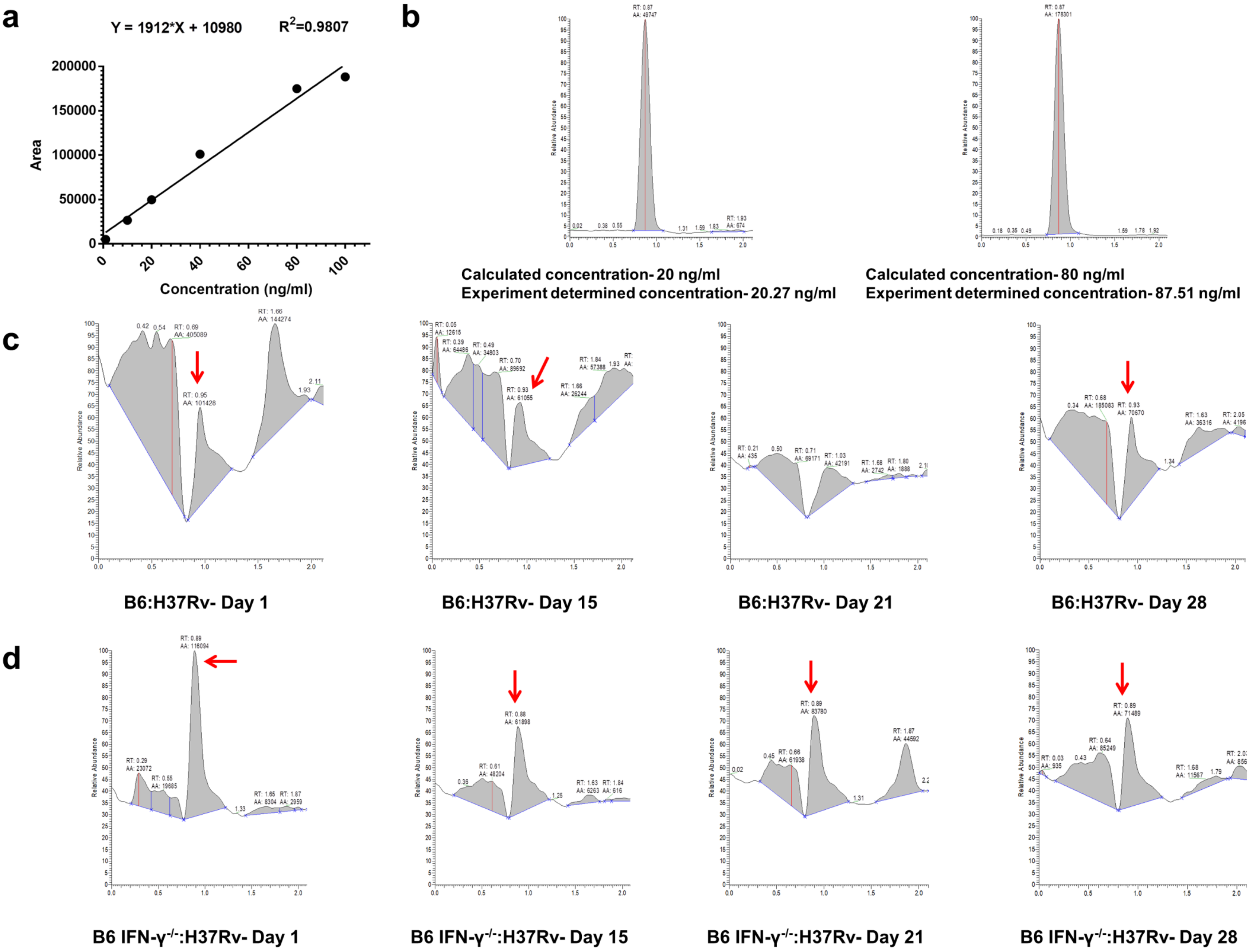
a
Y = 1912*X + 10980
R2=0.9807
Area
Concentration (ng/ml)
b
Calculated concentration- 20 ng/ml
Experiment determined concentration- 20.27 ng/ml
Calculated concentration- 80 ng/ml
Experiment determined concentration- 87.51 ng/ml
c
B6:H37Rv- Day 1
B6:H37Rv- Day 15
B6:H37Rv- Day 21
B6:H37Rv- Day 28
d
B6 IFN-γ-/-:H37Rv- Day 1
B6 IFN-γ-/-:H37Rv- Day 15
B6 IFN-γ-/-:H37Rv- Day 21
B6 IFN-γ-/-:H37Rv- Day 28

Supplementary Figure 21: Linearity and quality control analysis of SRM method development for intracellular free tryptophan quantitation in mice lung lysates. (a) Linearity curve generated for pure tryptophan; concentrations ranging from 1 to 100 ng ml^{-1}. **(b)** Quality control test for two different concentrations of pure tryptophan and their respective quantitation from the linearity plot suggesting a high degree of accuracy of the developed method and the linearity curve (x-axis represents time). **(c)** LC-MS peaks for free histidine in B6 mice lungs infected with H37Rv on day 1, day 15 and day 28. Tryptophan levels were below the level of detection on day 21 (Graphs are representative of 6 samples for each time point; x-axis represents time). **(d)** LC-MS peaks for free histidine in B6 IFN-$\gamma^{-/-}$ mice lungs infected with H37Rv on day 1, day 15, day 21 and day 28 (Graphs are representative of 6 samples for each time point; x-axis represents time).

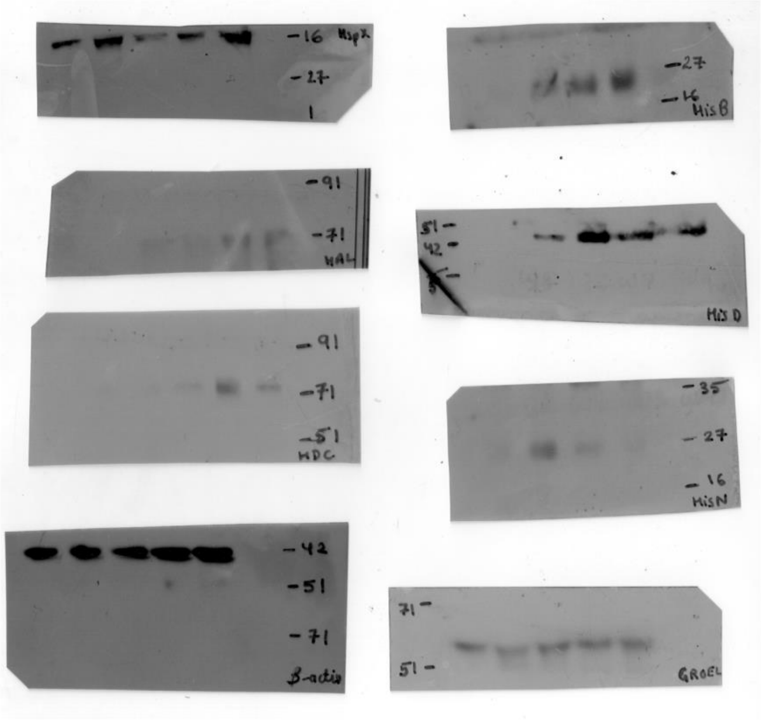

Mtb HspX
Mus HAL
Mus HDC
Mus β-actin

Mtb HisB
Mtb HisD
Mtb HisN
Mtb Groel2

Figure 2A

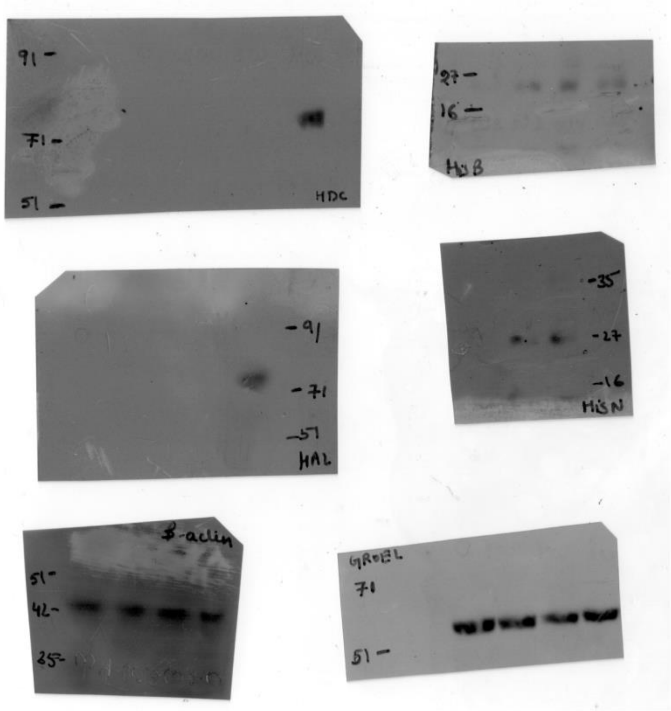

Mus HAL
Mus HDC
Mus β-actin

Mtb HisB
Mtb HisN
Mtb Groel 2

Figure 2B

Supplementary Figure 22: Unprocessed western blots for images presented in Figures 2A and B.

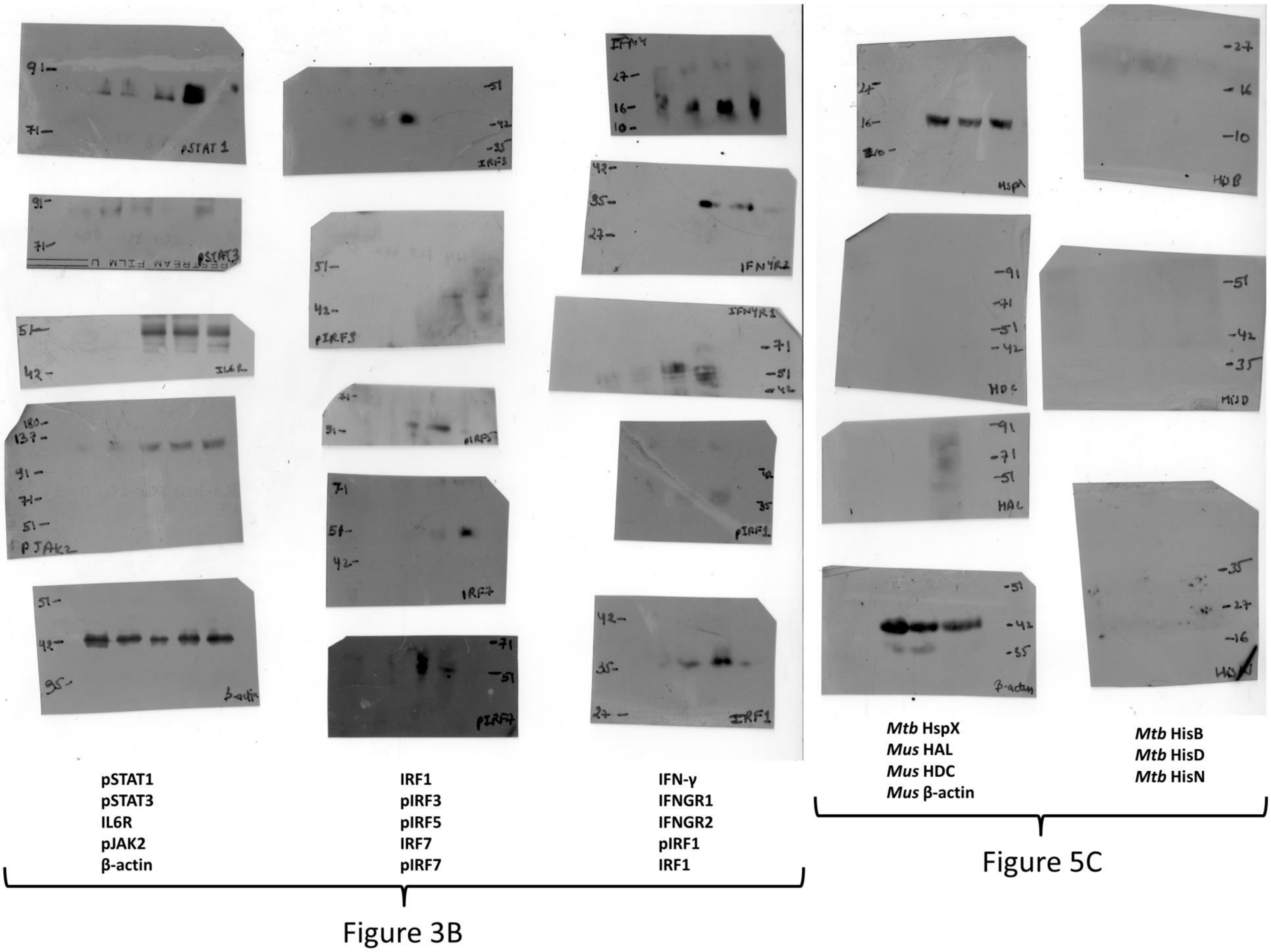

Supplementary Figure 23: Unprocessed western blots for images presented in Figures 3B and 5C.

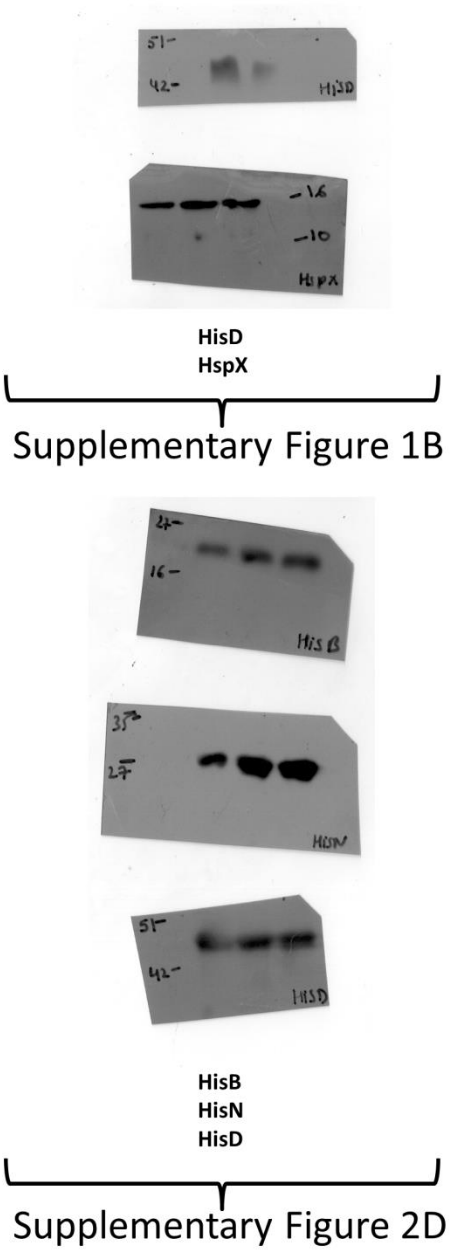

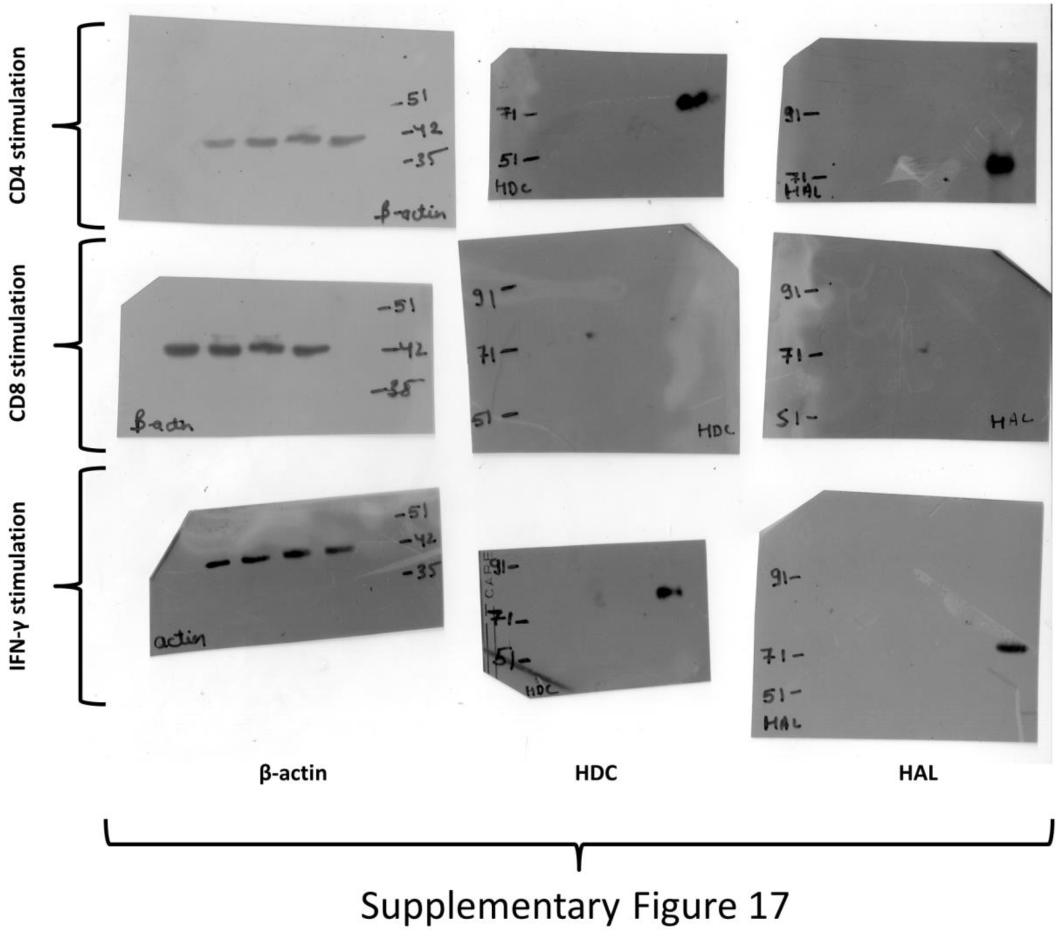

Supplementary Figure 24: Unprocessed western blots for images presented in Supplementary Figures 1B, 2D and 17.

SUPPLEMENTARY TABLES

Supplementary Table 1: Data Quality Summary for RNA Seq

Sample	Raw Reads	Clean Reads	Raw Base(G)	Clean Base(G)	Effective Rate(%)	Error Rate(%)	Q20(%)	Q30(%)	GC Content (%)
**Uninfected_1**	30460088	29768175	9.1	8.9	97.73	0.03	97.06	92.47	48.83
**Uninfected_2**	20215567	19913771	6.1	6	98.51	0.03	97.28	92.78	49.66
**Uninfected_3**	24176685	23714514	7.3	7.1	98.09	0.03	97.58	93.41	49.24
**Infected_1**	25003229	24267326	7.5	7.3	97.06	0.03	97.05	92.44	50.07
**Infected_2**	22673322	22048252	6.8	6.6	97.24	0.03	97.33	92.98	49.89
**Infected_3**	22673322	30022640	9.2	9	98.07	0.03	97.16	92.73	49.64

Supplementary Table 2: Data QC Statistics for RNA Seq

Sample	Infected_1	Infected_2	Infected_3	Uninfected_1	Uninfected_2	Uninfected_3
Reads (Raw)	50006458	45346644	61227726	60920176	40431134	48353370
Bases (Raw)	7.5E+09	6.8E+09	9.18E+09	9.14E+09	6.06E+09	7.25E+09
Reads (Clean)	49501560	44971046	60622602	60285282	40094372	47995746
Bases (Clean)	7.38E+09	6.7E+09	9.06E+09	9E+09	5.99E+09	7.17E+09
Q30 %(Clean)	92.84	93.32	93.13	92.88	93.11	93.7
Q30 bases (Clean)	6.85E+09	6.26E+09	8.43E+09	8.36E+09	5.58E+09	6.72E+09
GC% (Clean)	50.073	49.878	49.644	48.838	49.661	49.248

Supplementary Table 3: Read Alignment Summary for RNA Seq

Sample	Infected_1	Infected_2	Infected_3	Uninfected_1	Uninfected_2	Uninfected_3
paired_total	24750780	22485523	30311301	30142641	20047186	23997873
paired_aligned_none	1852661	1048968	1594840	1588743	835352	949104
paired_aligned_one	20998954	20005950	26205210	26382656	17769861	21230380
paired_aligned_multi	1868552	1410885	2484065	2145249	1422978	1795203
paired_aligned_discord_one	30613	19720	27186	25993	18995	23186
unpaired_total	3705322	2097936	3189680	3177486	1670704	1898208
unpaired_aligned_none	2628631	1339444	2047261	2022232	1006469	1183535
unpaired_aligned_one	998445	712743	1051862	1082897	619313	663185
unpaired_aligned_multi	78246	45749	90557	72357	44922	51488
overall_alignment_rate	94.69	97.02	96.62	96.65	97.49	97.53

Supplementary Table 4: Feature Assignment Summary for ReadCounts

Sample	Total	Assigned	Unassigned_Unmapped	Unassigned_NoFeatures	percent_assigned
**Infected_1**	27789678	24502365	1920563	1366750	88.17
**Infected_2**	24661478	22364708	1086692	1210078	90.69
**Infected_3**	34512999	31279901	1676014	1557084	90.63
**Uninfected_1**	33533795	30289283	1655864	1588648	90.32
**Uninfected_2**	22244334	20369426	872393	1002515	91.57
**Uninfected_3**	26937105	24380256	992939	1563910	90.51

Supplementary Table 5: List of Genes used to generate the interaction network in figure 3A

Ensembl Gene ID	Ensembl Protein ID	Entrez Gene ID	Gene Name	Log2 Fold Change	P Value	Molecule Type
ENSMUSG00000026638	ENSMUSP00000075839	54139	Irf6	-0.595516984	0.001733	TF
ENSMUSG00000031627	ENSMUSP00000147318	16363	Irf2	0.576392209	0.007855	TF
ENSMUSG00000025498	ENSMUSP00000147529	54123	Irf7	2.350829222	2.13E-30	TF
ENSMUSG00000003184	ENSMUSP00000147187	54131	Irf3	0.494472979	0.003267	TF
ENSMUSG00000018899	ENSMUSP00000122101	16362	Irf1	1.796486967	1.49E-26	TF
ENSMUSG00000029771	ENSMUSP00000145299	27056	Irf5	1.96456213	1.32E-20	TF
ENSMUSG00000021356	ENSMUSP00000105936	16364	Irf4	1.027068215	9.87E-04	TF
ENSMUSG00000002325	ENSMUSP00000120525	16391	Irf9	0.74204397	4.36E-05	TF
ENSMUSG00000041515	ENSMUSP00000125447	15900	Irf8	2.119757976	6.57E-14	TF
ENSMUSG00000055170	ENSMUSP00000063800	15978	Ifng	5.530293415	1.32E-45	Cytokine
ENSMUSG00000014599	ENSMUSP00000119553	12977	Csf1	0.448188255	0.008152	Cytokine
ENSMUSG00000078853	ENSMUSP00000130878	16145	Igtp	3.597084485	5.54E-89	GTPase
ENSMUSG00000054072	ENSMUSP00000063390	60440	Iigp1	3.442418737	1.04E-84	GTPase
ENSMUSG00000062939	ENSMUSP00000130713	20849	Stat4	0.061029833	0.700058	Signal_Transducer
ENSMUSG00000040033	ENSMUSP00000100872	20847	Stat2	0.497555368	0.012422	Signal_Transducer
ENSMUSG00000020009	ENSMUSP00000129309	15979	Ifngr1	-0.034775141	0.820549	Receptor
ENSMUSG00000022965	ENSMUSP00000119580	15980	Ifngr2	0.657393922	5.73E-04	Receptor
ENSMUSG00000026104	ENSMUSP00000141144	20846	Stat1	1.285561232	2.73E-11	Signal_Transducer
ENSMUSG00000002147	ENSMUSP00000112722	20852	Stat6	2.445097561	1.47E-45	Signal_Transducer
ENSMUSG00000031805	ENSMUSP00000105640	16453	Jak3	1.155520391	6.18E-07	Kinase
ENSMUSG00000028530	ENSMUSP00000122957	16451	Jak1	-0.137376473	0.408235	Kinase

ENSMUSG00000024621	ENSMUSP00000110923	12978	Csf1r	0.934273993	1.42E-06	Receptor
ENSMUSG00000024789	ENSMUSP00000064394	16452	Jak2	0.529891199	9.16E-04	Kinase
ENSMUSG00000025746	ENSMUSP00000143544	16193	Il6	4.967663023	5.37E-15	Cytokine
ENSMUSG00000021756	ENSMUSP00000139311	16195	Il6st	-2.310264327	0.001249	Signal_Transducer
ENSMUSG00000027947	ENSMUSP00000143541	16194	Il6ra	0.10079715	0.631017	Receptor
ENSMUSG00000004040	ENSMUSP00000121677	20848	Stat3	1.292914991	4.38E-05	Signal_Transducer
ENSMUSG00000024927	ENSMUSP00000025867	19697	Rela	0.298517538	0.113805	TF
ENSMUSG00000024401	ENSMUSP00000126122	21926	Tnf	2.86621029	0.021771	Cytokine
ENSMUSG00000020017	ENSMUSP00000123336	15109	Hal	2.088341917	4.10E-15	Enzyme
ENSMUSG00000027360	ENSMUSP00000028838	15186	Hdc	-0.19747295	0.314106	Enzyme

Supplementary Table 6: Reagent/Resource/Tools

**Reagent/Resource/Tools**	**Reference/Source**	**Identifier/ Catalog**
**Experimental animals, cell lines and bacterial strains**		
C57BL/6J (*M. musculus*)	Jackson Lab, USA	000664
C57BL/6J MHC-II KO(*M. musculus*)	Jackson Lab, USA	003584
C57BL/6J IFN-γ KO(*M. musculus*)	Jackson Lab, USA	002287
New Zealand White (*O. cuniculus*)	Charles River	571 (Oakwood)
H37Rv (*M. tuberculosis*)	ATCC, USA	27294
H37Rv Rv1599 KO (*M. tuberculosis*)	Laboratory of Dr. Tanya Parish, USA	NA
H37Rv *msh1* KD (*M. tuberculosis*)	Singh et. al., 2017, *Journal of Biological Chemistry*	NA
mc^2-4517 (*M. smegmatis*)	Laboratory of Prof. William R. Jacobs, USA	NA
DH5α (*E. coli*)	ThermoFisher, USA	18258012
Raw 264.7	ATCC, USA	TIB-71
Thp 1	ATCC, USA	TIB-202
Primary macrophages	This study	NA
CD4 T cells	This study	NA
CD8 T cells	This study	NA

**Recombinant DNA, oligonucleotides, recombinant proteins and enzymes**		
pYUB-1062	Laboratory of Prof. William R. Jacobs, USA	NA
pNIT1	Pandey et. al., 2009, *Tuberculosis*	NA
pENTR-D-TOPO	Invitrogen, USA	
Genomic DNA (*M. tuberculosis*)	BEI Resources, USA	NR-48669
H37Rv Rv1600 expression primers	Ahangar et. al., 2011, *Acta Crystallographica Section F*	NA
H37Rv Rv1599 expression primers	This Study, See Methods and Protocols	NA
H37Rv Rv3137 expression primers	Jha et. al., 2018, *The Journal of biological chemistry*	NA
H37Rv HisB	Ahangar et. al., 2011, *Acta Crystallographica Section F*	NA
H37Rv HisD	This Study	NA
H37Rv HisN	Jha et. al., 2018, *The Journal of biological chemistry*	NA
Restriction endonuclease NdeI	New England Biolabs	R0111L
Restriction endonuclease HindIII	New England Biolabs	R0104L
T4 DNA Ligase	New England Biolabs	M0202L
**Antibodies, chemicals and reagents**		
Rabbit anti- *Mtb* HisB	This study	NA
Rabbit anti- *Mtb* HisD	This study	NA

Rabbit anti- *Mtb* HisN	This study	NA
Rabbit anti- *Mtb* HspX	BEI Resources, USA	NR-13607
Rabbit anti- *Mtb* Groel2	BEI Resources, USA	NR-13655
Rabbit anti- Mice HAL	ThermoFisher, USA	PA5-88922
Rabbit anti- Mice HDC	ThermoFisher, USA	PA5-79354
Rabbit anti- Mice IFN-γ	ThermoFisher, USA	710150
Rabbit anti- Mice IRF1	ThermoFisher, USA	A303-378A
Rabbit anti- Mice IRF3	ThermoFisher, USA	A303-384A
Rabbit anti- Mice IRF7	ThermoFisher, USA	PA5-20281
Rabbit anti- Mice pSTAT1	ThermoFisher, USA	44-376G
Rabbit anti- Mice pSTAT3	ThermoFisher, USA	44-384G
Rabbit anti- Mice IFN-γ receptor 1	ThermoFisher, USA	12-1191-82
Rabbit anti- Mice IFN-γ receptor 2	R&D Systems, USA	MAB773
Rabbit anti- Mice pIRF1	ThermoFisher, USA	8478S
Rabbit anti- Mice pIRF3	Abbexa, UK	abx012441
Rabbit anti- Mice pIRF5	Abbexa, UK	abx216307
Rabbit anti- Mice pIRF7	Abbexa, UK	abx216310

Rabbit anti- Mice pJAK2	Abbexa, UK	abx333302
Rabbit anti- Mice IL-6 receptor	ThermoFisher, USA	AHR0061
Goat anti-Rabbit IgG secondary antibody	ThermoFisher, USA	G-21234
L-1-methylhistidine	SigmaAldrich, USA	67520
5-methyl-1-H-imidazole	TCI, India	M2228
L-histidine hydroxamate	SigmaAldrich, USA	H8500
D-histidine	SigmaAldrich, USA	H3751
Ficoll	Himedia, India	LS005
GM-CSF	Himedia, India	TC315
IFN-γ	SigmaAldrich, USA	11276905001
RPMI-1640	Himedia, India	AL028A
Fetal bovine serum	Himedia, India	RM9955
Trypsin - EDTA	Himedia, India	TCL007
Neomycin	Himedia, India	CMS214
Penicillin-Streptomycin	Himedia, India	A018
Kanamycin	Himedia, India	MB105
Hygromycin	Himedia, India	CMS2573

Anhydro-tetracycline	SigmaAldrich, USA	37919
Middlebrook 7H9 broth	Himedia, India	M198
Middlebrook 7H10 agar	Himedia, India	M199
OADC	Himedia, India	FD018
ADC	Himedia, India	FD019
Luria–Bertani Broth	Himedia, India	M1245
Luria–Bertani Agar	Himedia, India	M1151
3,3',5,5'-tetramethylbenzidine	Himedia, India	MB122
Freund's incomplete adjuvant	SigmaAldrich, USA	F5506
Protease Inhibitor Cocktail	SigmaAldrich, USA	S8820
**Software, resources, technical kits etc**		
RNA Seq data processing and analysis	See Supplementary Table 7	NA
LC MS/MS data processing and analysis	Thermo Trace Finder & Thermo Xcalibur	NA
Statistical Analysis	Graphpad Prism 7	NA
Nucleotide sequencing	Macrogen Ltd., USA	NA
RNA Seq	Nucleome Informatics, Hyderabad, India	NA
LC MS/MS	VProteomics, New Delhi, India	NA

CD4 Selection Kit	Miltenyi Biotec, USA	130-104-454
CD8 Selection Kit	Miltenyi Biotec, USA	130-104-075
Cell Disruptor	Constant Systems, UK	CF1
Ni-NTA His Trap column	GE Healthcare, USA	17524801
HiLoad 16/600 Superdex 200 pg column	GE Healthcare, USA	28989335
Electroporation Cuvette	BioRad, USA	1652082
Centricon	Millipore, USA	UFC903024, UFC800324, MRCPRT010
ELISA Kit	ThermoFischer, USA	NA

Supplementary Table 7: Softwares and Parameters for RNA Seq data processing and analysis

Analysis	Software	Parameters
QC	fastp	-w 8 -M 20 -W 4 -5 -3 -l 50 -z 5
Alignment	Hisat2	default
Assign features	FeatureCounts	-g transcript_id/gene_id -M -O -p -B -T 10 -a
DEG	DESeq2	|log2FC|>2, padj <0.05 , FDR correction
KEGG	KEGGParser, KEGGScape; Cytoscape	Padj<0.005, overrepresentation, FWER
Reactome	Reactome FI; Cytoscape	Padj<0.005, overrepresentation, FDR
Wiki Pathways	WikiPathways; Cytoscape	Padj<0.05, overrepresentation
Network Generation	GeneMANIA, Diffany; Cytoscape	Padj<0.05, Regulation
Ontology	ClueGO; Cytoscape	Padj<0.005, overrepresentation, FDR
