# Supplementary material for: De novo histidine biosynthesis protects Mycobacterium tuberculosis from host IFN-γ mediated histidine starvation
Source: Commun Biol. 2021 Mar 25;4:410. doi: 10.1038/s42003-021-01926-4 (PMC7994828; doi:10.1038/s42003-021-01926-4)
Supplement: Supplementary file 3 — Description of Additional Supplementary Files [file 42003_2021_1926_MOESM3_ESM.pdf]

Description of Additional Supplementary Files

File Name: Supplementary Data 1

Description: List of normalized readcounts

File Name: Supplementary Data 2

Description: List of differentially expressing genes infected vs uninfected.

File Name: Supplementary Data 3

Description: GO Immune Functions populated with up and down regulated genes obtained from the RNAseq analysis.

File Name: Supplementary Data 4

Description: Source data for the graphs presented in Main and Supplementary figures.
